# Discovery of 1-(5-bromopyrazin-2-yl)-1-[3-(trifluoromethyl)benzyl]urea as a promising anticancer drug via synthesis, characterization, biological screening, and computational studies

**DOI:** 10.1038/s41598-023-44662-x

**Published:** 2023-12-20

**Authors:** Yasser Hussein Issa Mohammed, Israa M. Shamkh, Nahed S. Alharthi, Mohammed A. Shanawaz, Hind A. Alzahrani, Basit Jabbar, Saba Beigh, Saad Alghamdi, Nada Alsakhen, Elshiekh B. Khidir, Hayaa M. Alhuthali, Taqwa Hafiz Elamin Karamalla, Amgad M. Rabie

**Affiliations:** 1 Department of Biochemistry, Faculty of Applied Science, University of Hajjah, Hajjah, Yemen; 2 Department of Pharmacy, Faculty of Medicine and Medical Science, University of Al-Razi, Sana’a, Yemen; 3 Botany and Microbiology Department, Faculty of Science, Cairo University, Giza, Egypt; 4 Chemo and Bioinformatics Lab, Bio Search Research Institution (BSRI), Giza, Egypt; 5 Department of Medical Laboratory Sciences, College of Applied Medical Sciences in Al-Kharj, Prince Sattam Bin Abdulaziz University, Al-Kharj, 11942 Saudi Arabia; 6 Department of Public Health, Faculty of Applied Medical Sciences, Albaha University, Albaha, 65431 Saudi Arabia; 7 Department of Basic Sciences, Faculty of Applied Medical Sciences, Albaha University, Albaha, 65431 Saudi Arabia; 8 Centre of Excellence in Molecular Biology, University of the Punjab, Lahore, 53700 Pakistan; 9 Department of Clinical Laboratory Sciences, Faculty of Applied Medical Sciences, Umm Al-Qura University, Makkah, Saudi Arabia; 10 Department of Chemistry, Faculty of Science, The Hashemite University, Zarqa, Jordan; 11 Department of Clinical Laboratory Sciences, College of Applied Medical Sciences, Taif University, P.O. Box 11099, Taif, 21944 Saudi Arabia; 12 Faculty of Medicine, Helwan University, Helwan, Cairo Egypt; 13 Head of Drug Discovery and Clinical Research Department, Dikernis General Hospital (DGH), Magliss El-Madina Street, Dikernis City 35744, Dikernis, Dakahlia Governorate Egypt

**Keywords:** Biochemistry, Biological techniques, Biophysics, Cancer, Cell biology, Chemical biology, Computational biology and bioinformatics, Drug discovery, Immunology, Molecular biology, Physiology, Structural biology, Systems biology, Diseases, Health care, Medical research, Molecular medicine, Oncology, Chemistry

## Abstract

Cancer and different types of tumors are still the most resistant diseases to available therapeutic agents. Finding a highly effective anticancer drug is the first target and concern of thousands of drug designers. In our attempts to address this concern, a new pyrazine derivative, 1-(5-bromopyrazin-2-yl)-1-[3-(trifluoromethyl)benzyl]urea (**BPU**), was designed via structural optimization and synthesized to investigate its anticancer/antitumor potential. The in-vitro anticancer properties of **BPU** were evaluated by MTT assay using selected cell lines, including the Jurkat, HeLa, and MCF-7 cells. The Jurkat cells were chosen to study the effect of **BPU** on cell cycle analysis using flow cytometry technique. **BPU** exhibited an effective cytotoxic ability in all the three cell lines assessed. It was found to be more prominent with the Jurkat cell line (IC_50_ = 4.64 ± 0.08 µM). When it was subjected to cell cycle analysis, this compound effectively arrested cell cycle progression in the sub-G1 phase. Upon evaluating the antiangiogenic potential of **BPU** via the in-vivo/ex-vivo shell-less chick chorioallantoic membrane (CAM) assays, the compound demonstrated very significant findings, revealing a complementary supportive action for the compound to act as a potent anticancer agent through inhibiting blood vessel formation in tumor tissues. Moreover, the docking energy of **BPU** computationally scored − 9.0 kcal/mol with the human matrix metalloproteinase 2 (MMP-2) and − 7.8 kcal/mol with the human matrix metalloproteinase 9 (MMP-9), denoting promising binding results as compared to the existing drugs for cancer therapy. The molecular dynamics (MD) simulation outcomes showed that **BPU** could effectively bind to the previously-proposed catalytic sites of both MMP-2 and MMP-9 enzymes with relatively stable statuses and good inhibitory binding abilities and parameters. Our findings suggest that the compound **BPU** could be a promising anticancer agent since it effectively inhibited cell proliferation and can be selected for further in-vitro and in-vivo investigations. In addition, the current results can be extensively validated by conducting wet-lab analysis so as to develop novel and better derivatives of **BPU** for cancer therapy with much less side effects and higher activities.

## Introduction

Regardless of critical advances in diagnosis and treatment, malignancy is as yet an existence debilitating illness around the globe. Late examinations have concentrated on chemopreventive mixes having anticancer potential^1–3^. The control of cell expansion is one of the crucial key elements amid the association of multicellular living beings^3,4^. Absence of capacity to control cell multiplication prompts tumor. Apoptosis is a modified cell demise, and it happens regularly in the tissues amid advancement as a piece of homeostasis to manage development and also aging^4^. Numerous hostile-to-tumor specialists incite apoptosis to achieve remedial strength in cancer treatment^4–8^.

In the world of medicinal chemistry, a huge number of potent heterocyclic compounds are obtained from synthetic and also from natural sources. The majority of these compounds contain one or more nitrogen atoms in their heterocyclic ring(s). Pyrazine is one of the important six-membered heterocyclic compounds with two nitrogens as heteroatoms^9^. In the recent times, pyrazine derivatives have been much focused by medicinal chemists, phytochemists, and pharmacologists due to their wide range of biological activities^9^. Various structural alterations and substitutions were experimented with in the pyrazine ring system^9,10^. These structural modifications positively affected some productive biological activities of the compounds^9,10^. For instance, some promising analogs of pyrazine, isoxazolo[4,5-*d*]pyridazin-4(5*H*)-ones, were shown to possess good potent antiinflammatory activities^11^. Some other analogs and derivatives of pyrazine were shown to affect different cardiovascular diseases^12^. Similarly, a considerable number of pyrazine compounds have been reported to possess various important biological properties, including anticoronavirus disease 2019 (anti-COVID-19), antiviral, antimicrobial, antiplatelet aggregation, anticancer, antihypertensive, antidiabetic, and other several activities^10,13^.

**Figure 1:**
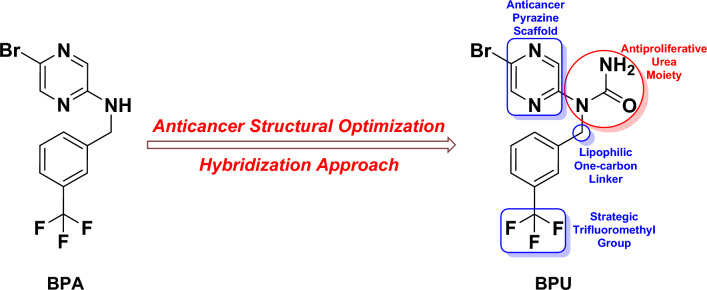
Chemical structures of the newly-discovered potential anticancer agent **BPU** and its computationally-suggested parent compound **BPA**. The figure also demonstrates the rational design (the key structural elements) of the hybridized molecule **BPU** obtained after structural modification of the parent molecule **BPA** to uncover the antitumor potentials.

More specifically, recent studies reported that some pyrazine derivatives bearing an amido/amino substitution at the 2/3-position possess potent to very potent in-vitro and in-vivo antitumor activities^14^. In this line of continuation, we did our best searching for new anticancer pyrazine candidates having the previous characteristics, using primarily predictive combinatorial data and computational optimization techniques. These several preparatory speculative steps have afforded a newly-proposed pyrazine molecule, 5-bromo-*N*-[3-(trifluoromethyl)benzyl]pyrazin-2-amine (**BPA; **Fig. 1), to be tried for having a significant anticancer potential. We have chemically synthesized the compound **BPA** and examined the potency of its anticancer activities. Unfortunately, this computationally-proposed compound showed weak activities in cancer cell lines. However, we did not lose hope, and these results incited us to discuss the possibility of structurally modifying **BPA**, e.g., through the addition of a small chemical substituent (moiety) that is previously well known to have anticancer effects, to improve these weak anticancer activities in newly-developed derivatives. One of the ideas was to convert the small secondary amino group in **BPA** into the wider urea moiety that has a free primary amino group at its unsubstituted end. This is because pyrazines bearing a urea moiety have been reported to exhibit excellent antiproliferative activities^15^. Based on this fact (hybridization of pyrazine scaffold with urea substituent) along with applying computational structural optimization techniques, we have designed a new hybrid pyrazine-urea molecule, 1-(5-bromopyrazin-2-yl)-1-[3-(trifluoromethyl)benzyl]urea (**BPU; **Fig. 1), and successfully synthesized/characterized it in order to investigate the improved antiproliferative and anticancer potential of this suggested novel pyrazine derivative against various cancer cell lines (mainly) in the present study. In addition, the antitumor biological screening was followed by extensive computational studies.

## Results and discussion

### Rationale and molecular design optimization

As previously mentioned in the “Introduction” section and as previously illustrated in Fig. 1, the compound **BPU** was obtained following the optimization efforts for the **BPA** structure to produce more potent anticancer effects. The fundamental rationale was to design classical pyrazine antitumors with improved modern substituted nitrogenous scaffolds, for instance, novel substituted-aminopyrazines and, later, novel pyrazinyl ureas. The electron-rich nature of the pyrazine ring is very favorable in cancer chemotherapy, specially if it is supported, either via direct or indirect substitution, with groups/moieties known to possess certain excellent degrees of anticancer actions like the bromine atom, amino group, urea moiety, and trifluoromethyl moiety^16,17^.

This dense electronic cloud (i.e., this relatively high electronegativity) of the pyrazine nucleus and its previously-mentioned linked substituents (as in the compound **BPU**) is very essential for the significant increase of the net antimetastatic and apoptotic activities of the whole designed anticancer molecule since it greatly boosts the inhibiting capacities on the different molecular mechanisms and cellular pathways of cancer progression as well as greatly enhances the hitting potentials against the diverse enzymes/proteins involved in inducing cancer progression^16,17^. It is also very important to know that replacing the amino group of the **BPA** molecule with a urea moiety in the **BPU** molecule greatly aids in obtaining a stable compound (a resonance-stabilized molecule) via the amide-iminol tautomerism. The aforementioned facts are supported by tens of literature reports that demonstrate the importance of natural and synthetic pyrazines and their analogs (i.e., and mainly their urea derivatives, as in the current case) as very pivotal structural motifs with extremely potent anticancer pharmacological and clinical activities at the subnanomolar levels (reaching as low as 0.01 nM in some cases), e.g., active natural products like cephalostatins and ritterazines and active synthetic compounds like AZD-8835 and CX-5461^13–17^.

### Synthesis and molecular structure elucidation

**Figure 2:**
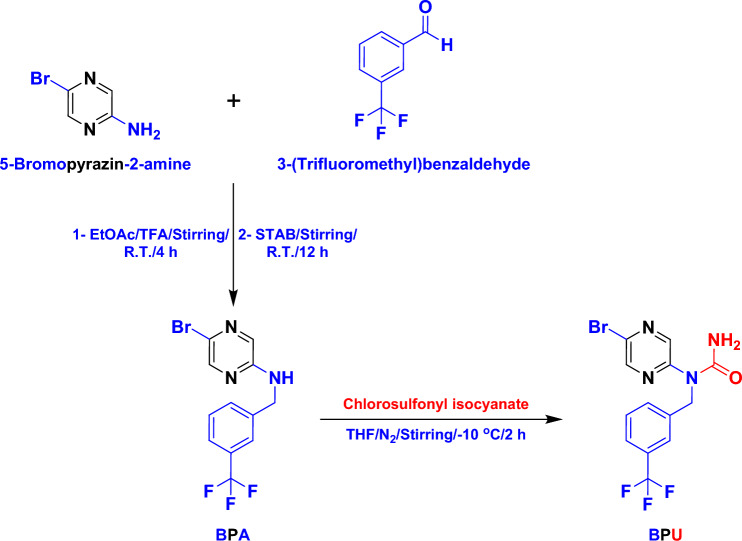
Schematic representation for the synthesis of the newly-designed potential anticancer agent **BPU**.

Preparation of the compound **BPA** was successfully accomplished via simple condensation of the primary amine 5-bromopyrazin-2-amine with the substituted benzaldehyde 3-(trifluoromethyl)benzaldehyde in excess ethyl acetate and trifluoroacetic acid (TFA) at room temperature (R.T.), followed by the addition of the reagent sodium triacetoxyborohydride (STAB) to the reaction mixture after 4 h at the same temperature until reaction completion. After purification of the crude **BPA** powder (excellent yield of 92%), it was used for the synthesis of the final compound **BPU** via substitution of the secondary amino proton using the dropwise addition of chlorosulfonyl isocyanate (with stirring) to a solution of **BPA** in THF at − 10 °C under nitrogen atmosphere. Thorough purification of the crude product afforded the pure **BPU** powder in a very good yield of 86% and an excellent purity of about 96% (checked by HPLC; see the “Supplementary Material file”). Both compounds are, by looking, almost freely soluble in dimethylsulfoxide (DMSO) and sparingly soluble in water. Figure 2 shows the representative synthetic scheme for **BPU** through **BPA**.

Elucidation and confirmation of the chemical structures of the two produced compounds, **BPA** and **BPU**, were successfully achieved via different spectroscopic and elemental analyses, as seen in the experimental part of this paper. Charts of the IR analysis showed peaks that correspond to the functional groups and principal moieties (e.g., C=N) in both compounds. This helps mainly to confirm the presence of the special fused C=N group of the aromatic pyrazine nucleus in the compounds **BPA** and **BPU**. On the other hand, ^1^H-NMR charts of the compounds **BPA** and **BPU** showed correct peaks for 9 and 10 protons, respectively; 8 of them represent the CH_2_, phenyl, and pyrazine protons in both compounds. The only distinct difference is the singlet peak that appears at 6.46 ppm in the **BPU** chart and is integrated into two protons, corresponding to the two protons of the NH_2_ group, while the analogous singlet peak in the **BPA** chart appears at 5.21 ppm and is integrated into only one proton, corresponding to the one proton of the NH group. ^13^C-NMR charts of the compounds **BPA** and **BPU** exactly showed 12 and 13 peaks whose chemical shifts specifically correspond to the 12 and 13 carbons of the two compounds, **BPA** and **BPU**, respectively. Charts of the mass spectral analysis showed molecular ion ([M]^+^) peaks at 332.02 (in the **BPA** chart) and 375.01 (in the **BPU** chart) as well as isotopic ion ([M + 2]^+^) peaks, of similar intensity to the relevant [M]^+^ peaks, at 334.15 (in the **BPA** chart) and 377.05 (in the **BPU** chart); these peaks exactly correspond to the molecular masses of the four isotopes (isotopes exist due to the presence of the bromine atom, which has two isotopes with a difference of 2 amu) of the two compounds, **BPA** and **BPU**, respectively. In addition, elemental analyses for the content percentages of the carbon, hydrogen, and nitrogen atoms in the samples of the two compounds were performed as a confirmation of the chemical structure of the compounds **BPA** and **BPU**. All the results of these elemental analyses were compatible with the proposed structures.

### Cell growth suppression analysis by MTT assay

After successful synthesis of the computationally-suggested new compound **BPA**, the compound was subjected to an in-vitro evaluation to determine its primary cytotoxic effectiveness in inhibiting the growth of cancer cells and also to decide if we would go far for further extensive investigative anticancer research on it or not. The results of the MTT assay in Table 1 showed that the compound **BPA** demonstrated a moderate level of cytotoxicity when tested against a specific cell line. This means that the compound was somewhat able to kill or inhibit the growth of some of the cells in that particular cell line. However, the current level of cytotoxicity was not strong enough to be considered a highly effective treatment for cancer (i.e., the anticancer activities of **BPA** are not sufficient to make it a potential antitumor drug to be used as a treatment option for cancer patients). As a result of this finding and since the ultimate goal of the current research is to develop a very potent drug that can be used to treat cancer patients, we decided not to continue to further anticancer research on **BPA** in its current chemical structure, and we also decided to explore the possibility of rationally modifying the chemical structure of **BPA** in order to improve its effectiveness against the different cancer cell lines. We planned to make very slight changes to the chemical composition of **BPA** (this might involve slightly altering certain molecular groups or adding small new ones to the compound to make it more effective) to see if they could increase its activity against cancer cells, as previously explained in the rational design part of the “Introduction” section. The first successful design attempt gave rise to the compound **BPU** (the major compound of the current work) as the first derivative of the parent compound **BPA**, opening the door for the establishment of this new series of potential anticancer drugs.

**Table 1:** Cytotoxic effects of the compound **BPA** on three human cancer cell lines in the MTT assay.

Cytotoxicity (IC_50_ in µM) of BPA against Several Cancer Cells^a^		
Sr. no.	Cell line type^b^	IC_50_ (µM) value^c^
1	MCF-7	29.10 ± 0.18
2	HeLa	32.84 ± 0.02
3	Jurkat	41.07 ± 0.11
4	NIH-3T3^d^	95.2 ± 0.12

^a^Cytotoxicity of **BPA** was measured by MTT assay against each cell line.

^b^The standard anticancer drugs (positive controls) used for the different cell lines were: Tamoxifen—MCF-7 cells; Avastin—HeLa cells; and Abitrexate—Jurkat cells. On the other hand, DMSO was used as a placebo drug (vehicle control), which showed very negligible cytotoxicity.

^c^Average IC_50_ values (expressed in µM) are indicated in plus or minus “ ± ” standard deviation (SD). Statistically significant values are expressed as *p < 0.05 and **p < 0.01.

^d^NIH/3T3 is a mouse embryonic fibroblast cell line that was isolated from a mouse NIH/Swiss embryo (i.e., cells that were derived from a Swiss mouse embryonic tissue). Another control with NIH-3T3 cells (with **BPA** administered) was run in parallel.

**Figure 3:**
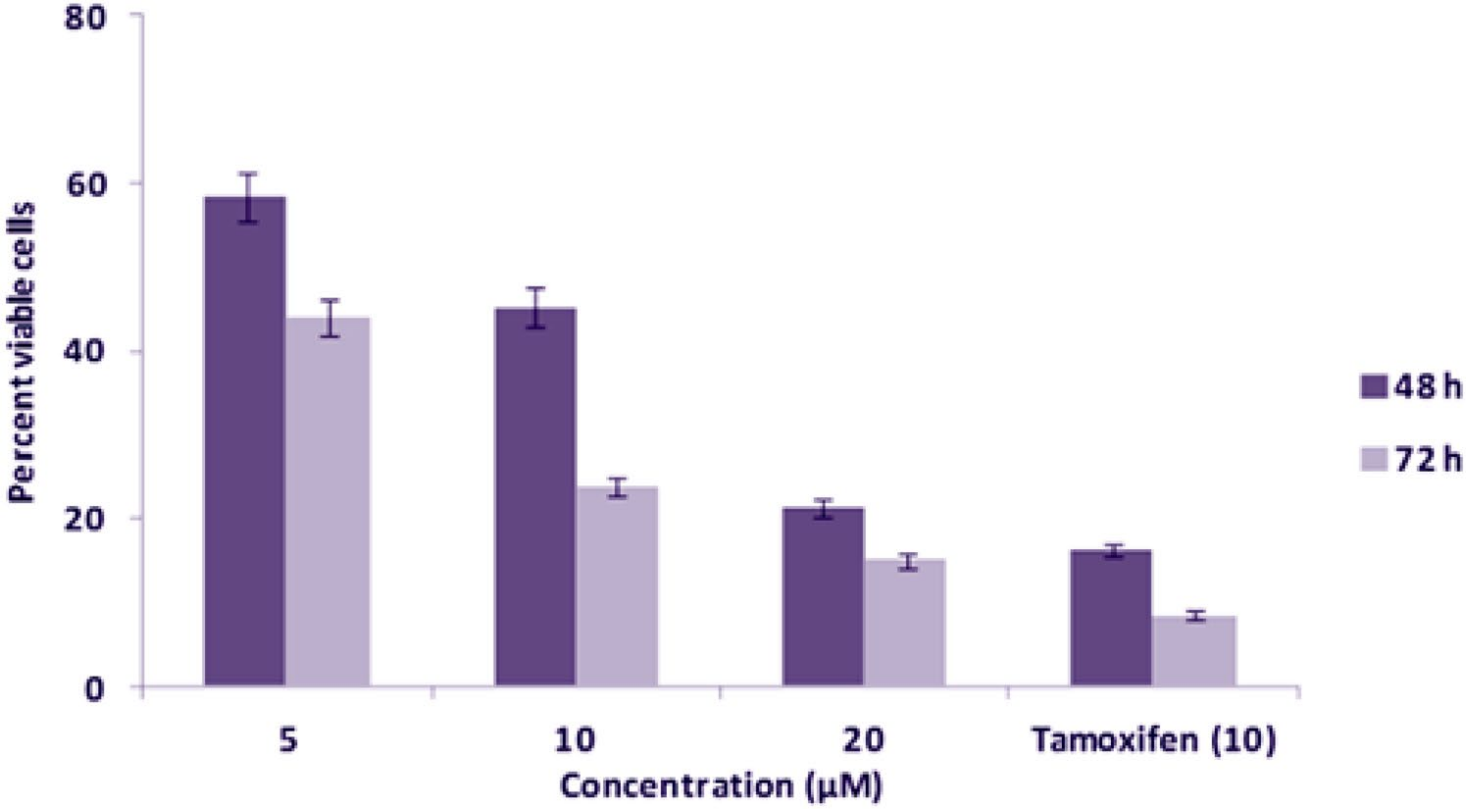
Evaluation results of cytotoxic effects of the compound **BPU** on MCF-7 cells by the MTT assay.

**Figure 4:**
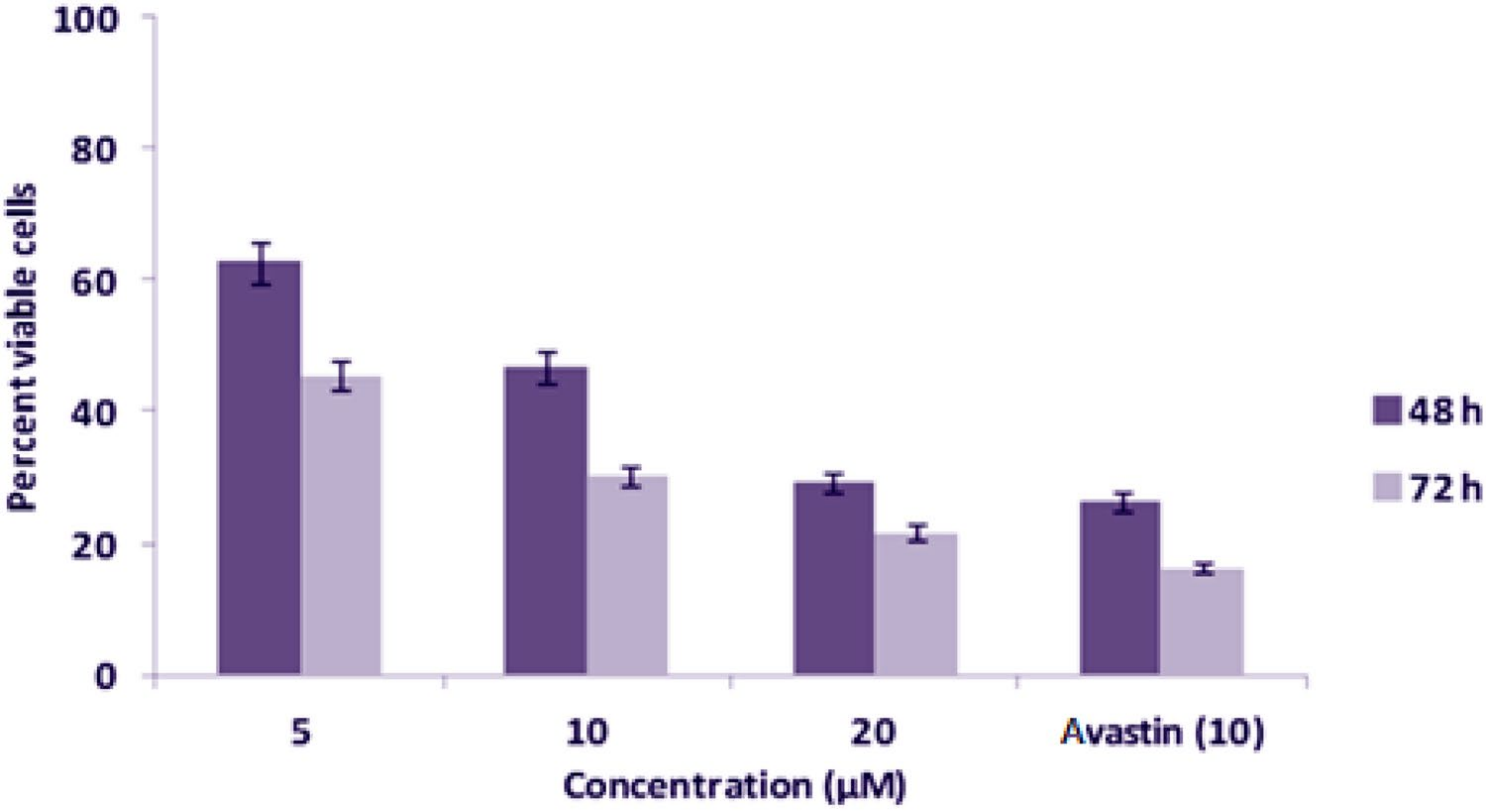
Evaluation results of cytotoxic effects of the compound **BPU** on HeLa cells by the MTT assay.

**Figure 5:**
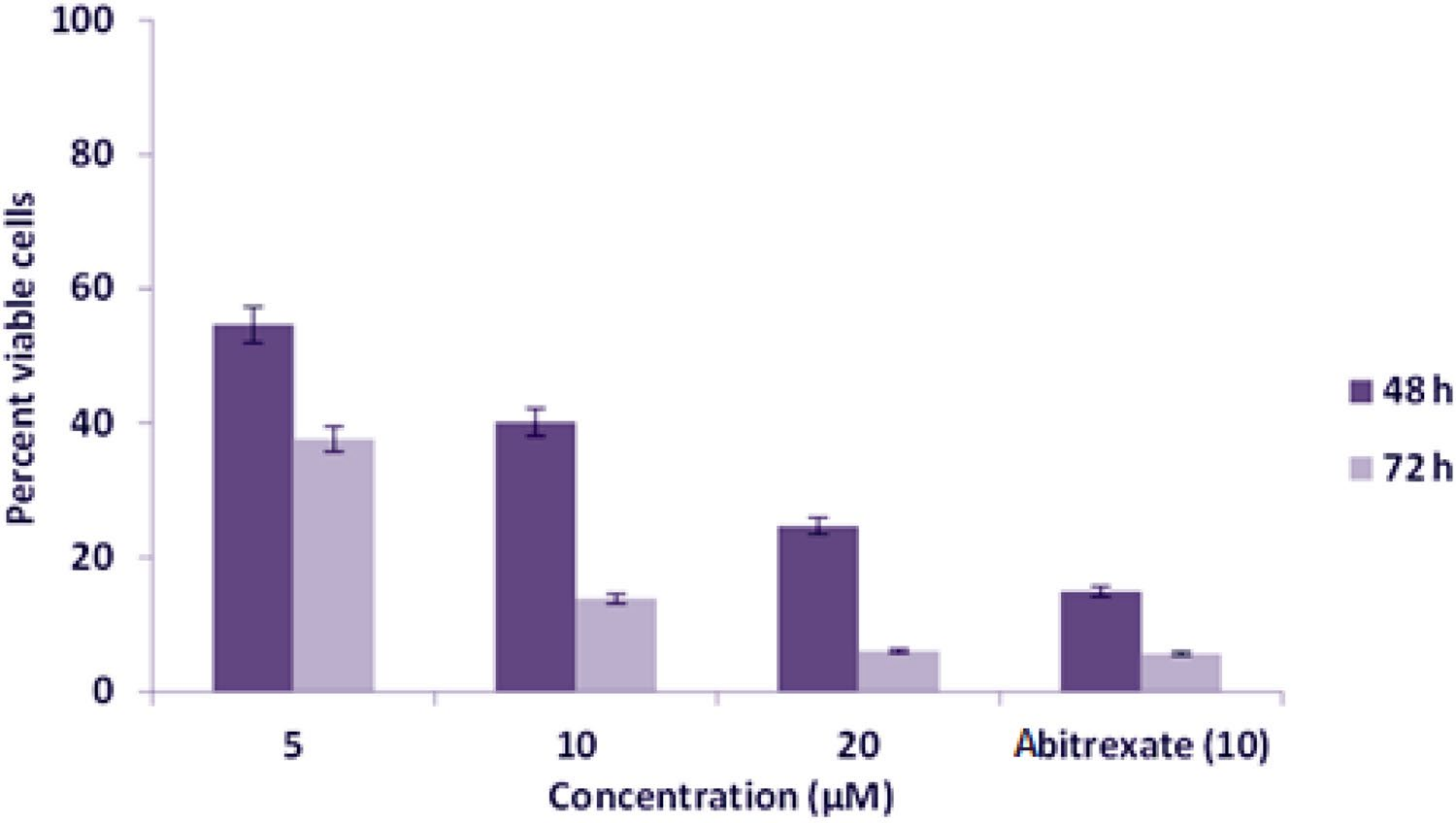
Evaluation results of cytotoxic effects of the compound **BPU** on Jurkat cells by the MTT assay.

The investigated target newly-synthesized compound **BPU** was diversely evaluated for the in-vitro cytotoxicity against three different cancer cell lines, MCF-7, HeLa, and Jurkat cells, by the MTT assay. The results of cytotoxicity studies of the compound **BPU** were recorded at different concentrations (5, 10, and 20 µM, respectively) and also at two different time intervals (48 and 72 h), as clearly illustrated in Figs. 3, 4, and 5, respectively, and summarized in Table 2.

**Table 2:** Cytotoxic effects of the compound **BPU** on three human cancer cell lines in the MTT assay.

Cytotoxicity (IC_50_ in µM) of BPU against Several Cancer Cells^a^		
Sr. no.	Cell line type^b^	IC_50_ (µM) value^c^
1	MCF-7	8.47 ± 0.18
2	HeLa	9.22 ± 0.17
3	Jurkat	4.64 ± 0.08
4	NIH-3T3^d^	89.8 ± 1.9

^a^Cytotoxicity of **BPU** was measured by MTT assay against each cell line.

^b^The standard positive controls used for the different cell lines were: Tamoxifen—MCF-7 cells; Avastin—HeLa cells; and Abitrexate—Jurkat cells. On the other hand, DMSO was used as a vehicle control, which showed very negligible cytotoxicity (as previously mentioned with evaluation of **BPA**).

^c^Average IC_50_ values (expressed in µM) are indicated in ± SD. Statistically significant values are expressed as *p < 0.05 and **p < 0.01.

^d^Another control with NIH-3T3 cells (with **BPU** administered) was run in parallel.

The data obtained from the MTT assay showed that the compound **BPU** has substantial inhibitory effects on the growth of MCF-7 cell lines after 48 and 72 h of treatment, and the IC_50_ value was found to be 8.47 ± 0.18 µM (Table 2). The percent viable cells at 5, 10, and 20 µM concentrations were found to be 58.48%, 45.22%, and 21.24%, respectively, after 48 h incubation. After 72 h incubation of the compound with the cells, the percent viable cells at 5, 10, and 20 µM concentrations were 43.89%, 23.88%, and 15.05%, respectively. The growth inhibitory effect of the compound **BPU** was found to increase at 72 h incubation. The standard anticancer drug Tamoxifen showed 16.36% and 8.56% of viable cells after 48 and 72 h incubation, respectively, at 10 µM concentration (Fig. 3). The high IC_50_ value of **BPU** against the NIH-3T3 cell lines (89.8 ± 1.9 µM) showed minimal effect of the drug on these cell lines, thus indicating the nontoxicity and relative safety of **BPU** against noncancerous and normal cells.

The compound **BPU** showed considerable inhibitory effects on the growth of HeLa cell lines after 48 and 72 h of treatment, and the IC_50_ value was found to be 9.22 ± 0.17 µM (Table 2). The percent viable cells at 5, 10, and 20 µM concentrations were found to be 62.67%, 46.77%, and 29.33%, respectively, after 48 h incubation. After 72 h incubation of the compound with the cells, the percent viable cells decreased to 45.53%, 30.38%, and 21.64% at 5, 10, and 20 µM concentrations, respectively. The growth inhibitory effect of the compound **BPU** significantly increased with increased incubation time. On the other hand, the standard anticancer drug Avastin allowed 26.38% and 16.33% of viable cells after 48 and 72 h incubation, respectively, at 10 µM concentration (Fig. 4).

Interestingly, the growth suppressive effect of the compound **BPU** was more prominent on Jurkat cells (the highest inhibition values), and inhibition was found to be more effective as compared to other cell lines studied. The data revealed by the MTT assay showed that the target compound **BPU** has significant inhibitory effects on the growth of Jurkat cell lines after 48 and 72 h of treatment. The IC_50_ value was highly significant and was found to be 4.64 ± 0.08 µM (Table 2). The percent viable cells at 5, 10, and 20 µM concentrations were observed to be 54.58%, 40.11%, and 24.72%, respectively, after 48 h incubation. After 72 h incubation of the compound with the cells, the percent viable cells at 5, 10, and 20 µM concentrations were 37.68%, 13.83%, and 6.01%, respectively (very apparent decrease in percent viable cells). The growth inhibitory effect of the compound **BPU** was considerably higher after 72 h of incubation compared to 48 h of incubation at equivalent concentrations of **BPU**. The standard anticancer drug Abitrexate showed 14.89% and 5.67% of viable cells after 48 and 72 h incubation, respectively, at 10 µM concentration (Fig. 5).

### Effect of the compound BPU on cell cycle progression

**Figure 6:**
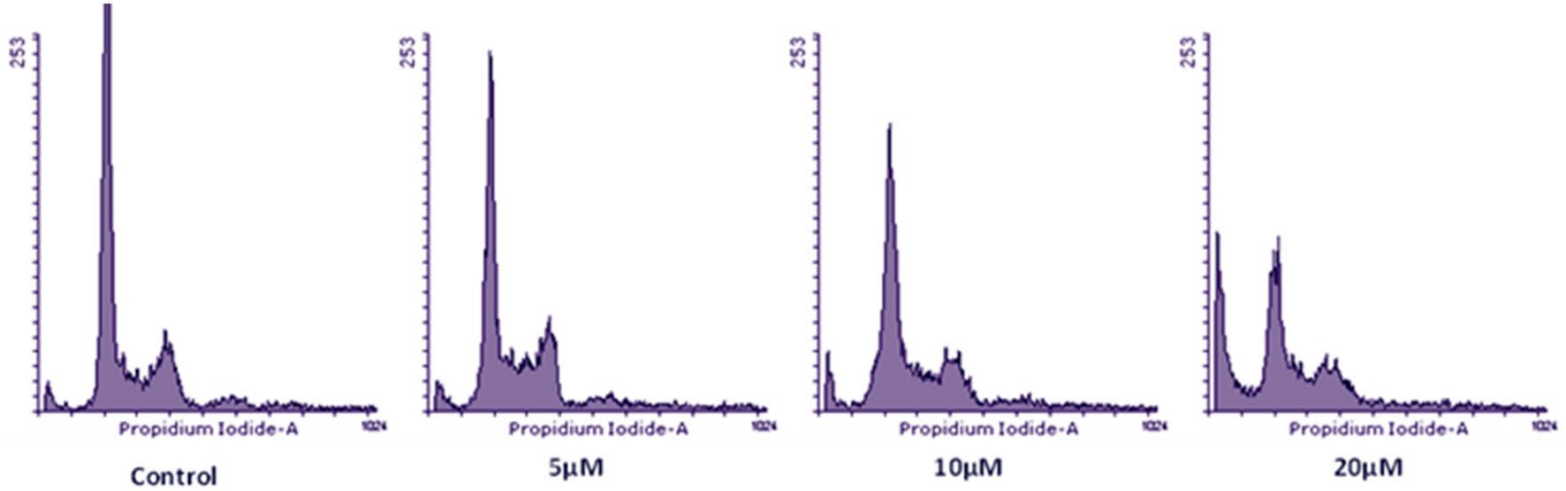
Histogram obtained after the FACS analysis for Jurkat cells (Control: DMSO-treated Jurkat cells; **BPU** effect was evaluated at different concentrations, 5/10/20 µM).

After preliminary studies of cytotoxicity through the MTT assay, the effect of **BPU** on cell cycle progression was evaluated by fluorescence-activated cell sorting (FACS). Similar concentrations of **BPU** were used in the FACS analysis as those used in the aforementioned cytotoxicity studies. Jurkat cells were chosen for the FACS analysis since they responded to the compound **BPU** treatment more significantly than the other two cell lines evaluated. The histogram of control cells treated with DMSO indicated a standard flow of cell cycle. Cell percentages in the different cell cycle phases of control cells were 63.80%, 11.54%, 4.30%, and 20.36% in the G1, S, sub-G1, and G2 phases, respectively. Cell cycle flow was significatively altered in different manners upon adding the compound **BPU** at different concentrations (Fig. 6).

**Figure 7:**
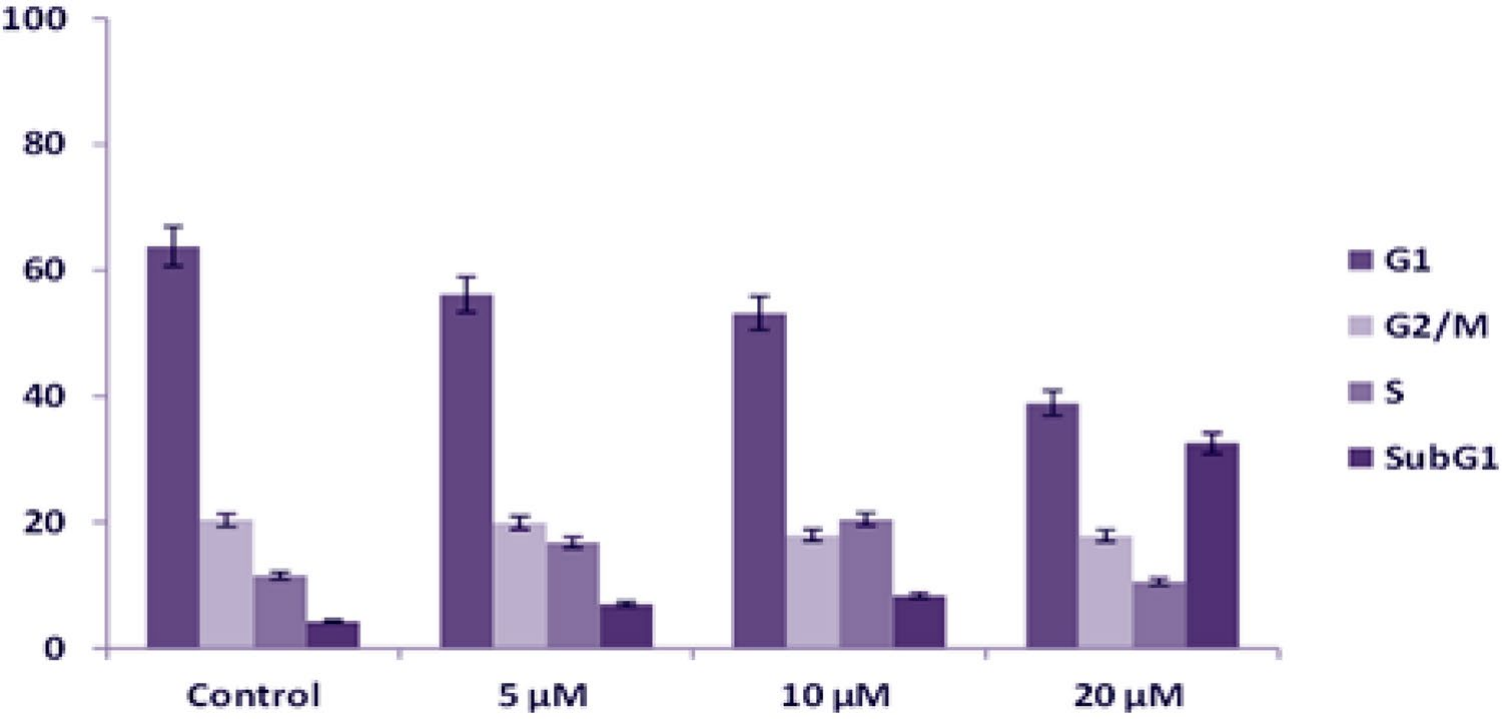
Bar graph presentation showing the percentage of cells (Y-axis) in the sub-G1, G1, S, and G2 phases of the cell cycle after the DMSO (control solvent)/**BPU** (target agent) treatments to evaluate the effect of the explored compound **BPU** (5, 10, and 20 µM concentrations, respectively) on cell cycle progression in Jurkat cells (after 48 h of treatment). The data shown were obtained from two independent experiments (at least) and error bars have been indicated.

The 5 and 10 µM concentrations of the compound **BPU** did not provoke any considerable effect on cell cycle progression. At the lowest concentration (5 µM) of the compound, the cell populations of the treated cells were found to be 56.19%, 16.84%, 7.03%, and 19.93% in the G1, S, sub-G1, and G2 phases, respectively. The cell distribution pattern in the 20-µM-**BPU**-treated Jurkat cells was found to be 38.95%, 10.56%, 32.57%, and 17.90% in the G1, S, sub-G1, and G2 phases, respectively (Figs. 6 and 7). At 20 µM concentration (for 48 h), the **BPU**-treated cells showed significant accumulation of cells in the sub-G1 phase (32.57%), which denotes prominence of this **BPU** concentration in arresting the cells in the sub-G1 phase. On the other side, the DMSO-treated Jurkat cells (control) exhibited a normal pattern of cell cycle with the highest cell population in the G1 phase (for 48 h). It is worth mentioning that heaping up of cells in the sub-G1 phase is an early indicator of apoptosis^18^. Furthermore, checkpoints in the cell cycle can also setback its progression or, in response to irreparable DNA damage, can cause induction of cell cycle exit or cell death^19^. These findings promisingly implied that the **BPU** treatment of Jurkat cells induced cell death which leads to accumulation of cells in the sub-G1 phase and apoptosis, thereby significantly affecting cells proliferation.

### Effect of the compound BPU on tissue angiogenesis

**Figure 8:**
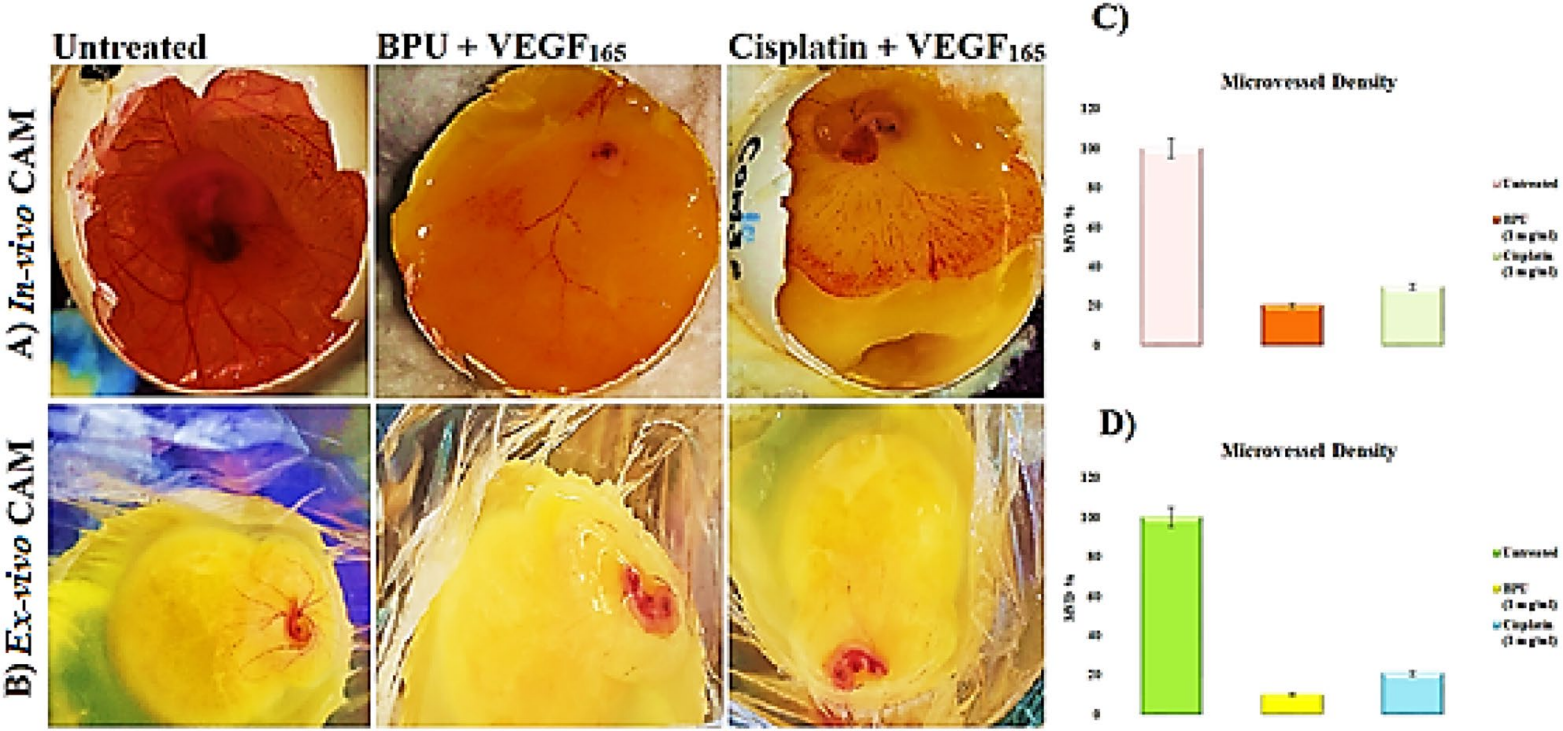
In-vivo/ex-vivo CAM photographs and bar graphs displaying the inhibition of neovessel formation as follows: (**A**) The in-vivo CAM photos exhibiting the angiopreventive effect in the **BPU**-treated CAM compared to that in the untreated (VEGF_165_ alone) and standard Cisplatin-VEGF_165_-treated CAMs. (**B**) The ex-vivo CAM photos exhibiting the angiopreventive effect in the **BPU**-treated CAM compared to that in the untreated (VEGF_165_ alone) and standard Cisplatin-VEGF_165_-treated CAMs. (**C**) Graphic representation of the microvessel density (MVD) counts in the in-vivo untreated, **BPU**-treated, and standard Cisplatin-treated CAMs. (**D**) Graphic representation of the MVD counts in the ex-vivo untreated, **BPU**-treated, and standard Cisplatin-treated CAMs. Statistically significant values are expressed as *p < 0.05 and **p < 0.01.

The compound **BPU** has been investigated in vivo for its potential to impede neovascularization, the process of new blood vessel formation, in noncancerous model systems, mainly using the in-vivo/ex-vivo shell-less chick chorioallantoic membrane (CAM) assays. The antiangiogenic activity of **BPU**, or its capability to inhibit the growth of new blood vessels, was studied across various angiogenesis models stimulated by the recombinant vascular endothelial growth factor-165-aa (rVEGF_165_ or VEGF_165_), a protein that promotes vascular growth. The number of sprouting vessels was measured using the control induced by rVEGF_165_ as the baseline (100%) in both in-vivo and ex-vivo CAM models. In a normal-developing CAM that was treated with only phosphate-buffered saline (PBS), it was observed that the growth of blood vessels amounted to 20% in both in-vivo and ex-vivo CAM assays. This serves as a contrast to the effects observed with the **BPU** treatment. Evident in both the in-vivo and ex-vivo CAM assays was a conspicuous avascular zone, or an area lacking blood vessels, with the **BPU** treatment. This suggests that **BPU** contributes to the regression, or reduction, of newly-formed vessels in the developing embryos. The inhibition rates were quite significant, measuring 83% and 77%, respectively. Moreover, there was a substantial decrease in the total length of the blood vessels in the eggs treated with **BPU**. The vessel length was reduced to approximately 92% of that observed in the untreated eggs in both assays, as indicated in Fig. 8A–D. The net effect of these results suggests that the compound **BPU** is a revolutionary agent for inhibiting blood vessel formation in noncancerous models and could be a potent inhibitor of tumor angiogenesis.

### Compound BPU shows promising predicted drug properties for cancer therapeutics

Numerous ADMET (adsorption, distribution, metabolism, excretion, and toxicity) properties were calculated for the **BPU** molecule, which indicated that it is soluble and safely passed the Lipinski's violations (Table 3); however, **BPU** computationally exhibited some acute oral toxicity (Table 4). Moreover, the docking and molecular dynamics (MD) simulations of **BPU** were carried out blindly with two closely-related proteins, the human matrix metalloproteinase 2 (MMP-2; also called gelatinase A) and the human matrix metalloproteinase 9 (MMP-9; also called gelatinase B), which are two of the important zinc-dependent endopeptidase proteins that are targetable in cancer therapy^20^, with the focus on the permanent strong interactions with the catalytic active sites and elemental ions of both of them. These two proteins were specifically chosen to be targeted in the current study because **BPU** is expected, based on the current biological findings, to combat different cancers by acting on and inhibiting mainly tumor growth and metastasis, like many structurally-similar compounds. The two matrix metalloproteinases (MMPs) are known for their critical roles in angiogenesis, tumor growth, and metastasis by degrading extracellular matrix and through the release and activation of various growth factors^20^. Such degradative activities result in progression of cancer through the enhancement of invasion and metastasis^20^. Therefore, effectively inhibiting both proteins, or at least one of them, is considered one of the most successful anticancer therapeutic pathways.

**Table 3:** Numerous physicochemical, ADMET, drug-likeness, and pharmacological properties of the compound **BPU**, estimated and predicted via the SwissADME server.

Property	Estimate/Prediction
Formula	C_13_H_10_BrF_3_N_4_O
MW	375.14
#Heavy atoms	22
#Aromatic heavy atoms	12
#Rotatable bonds	5
#H-bond acceptors	6
#H-bond donors	1
MR (molar refractivity)	76.72
TPSA (Å^2^)	72.11
Consensus Log *P*_o/w_	2.72
Ali class	Soluble
Log *S* (SILICOS-IT)	− 5.18
SILICOS-IT class	Moderately soluble
Log *K*_p_ (skin permeation; cm/s)	− 6.95
Lipinski #violations	0
Bioavailability score	0.55
GPCR (G protein-coupled receptor) ligand	0.31
Ion channel modulator	0.46
Kinase inhibitor	0.71
Nuclear receptor ligand	0.12
Protease inhibitor	0.19
Enzyme inhibitor	0.34

**Table 4:** Other ADMET properties and toxicity prediction of the compound **BPU**, estimated via the admetSAR 2.0 server.

Model	Estimate/Prediction	
BBB (blood–brain barrier)	Result	BBB +
	Probability	0.9814
HIA (human intestinal absorption)	Result	HIA +
	Probability	0.9839
Caco-2 permeability	Result	Caco-2 −
	Probability	0.5565
Carcinogenicity (three-class)	Result	Non-required
	Probability	0.5238
Acute oral toxicity	Result	III
	Probability	0.6057
Rat toxicity LD50 (mol/Kg)	Result	2.6653

**Figure 9:**
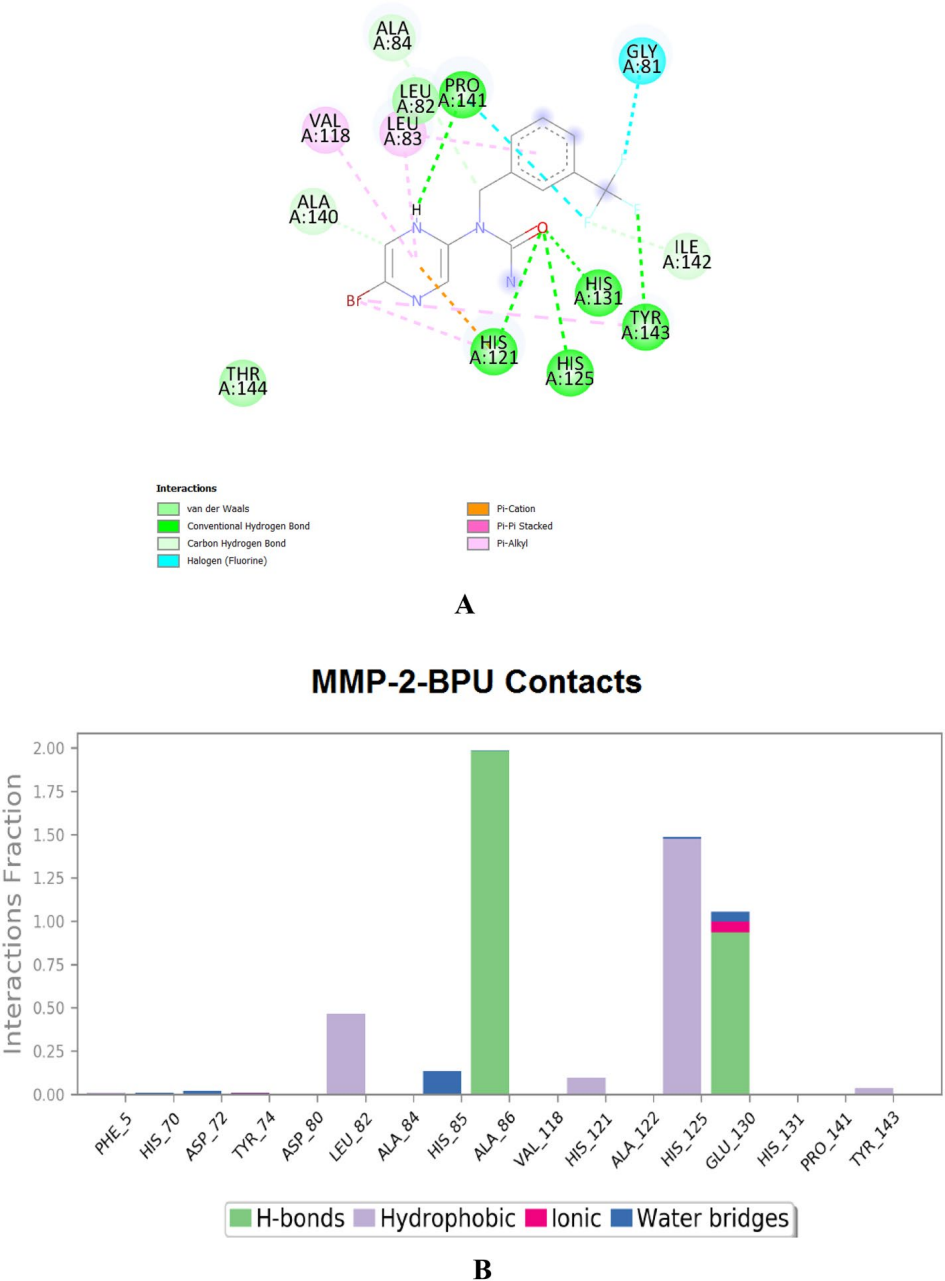
(**A**) Docking molecular interactions of the compound **BPU** with the protein MMP-2 (PDB ID: 7XGJ); net binding energy = − 9.0 kcal/mol. (**B**) MMP-2-**BPU** interactions fractions estimated by 100-ns MD simulation.

**Figure 10:**
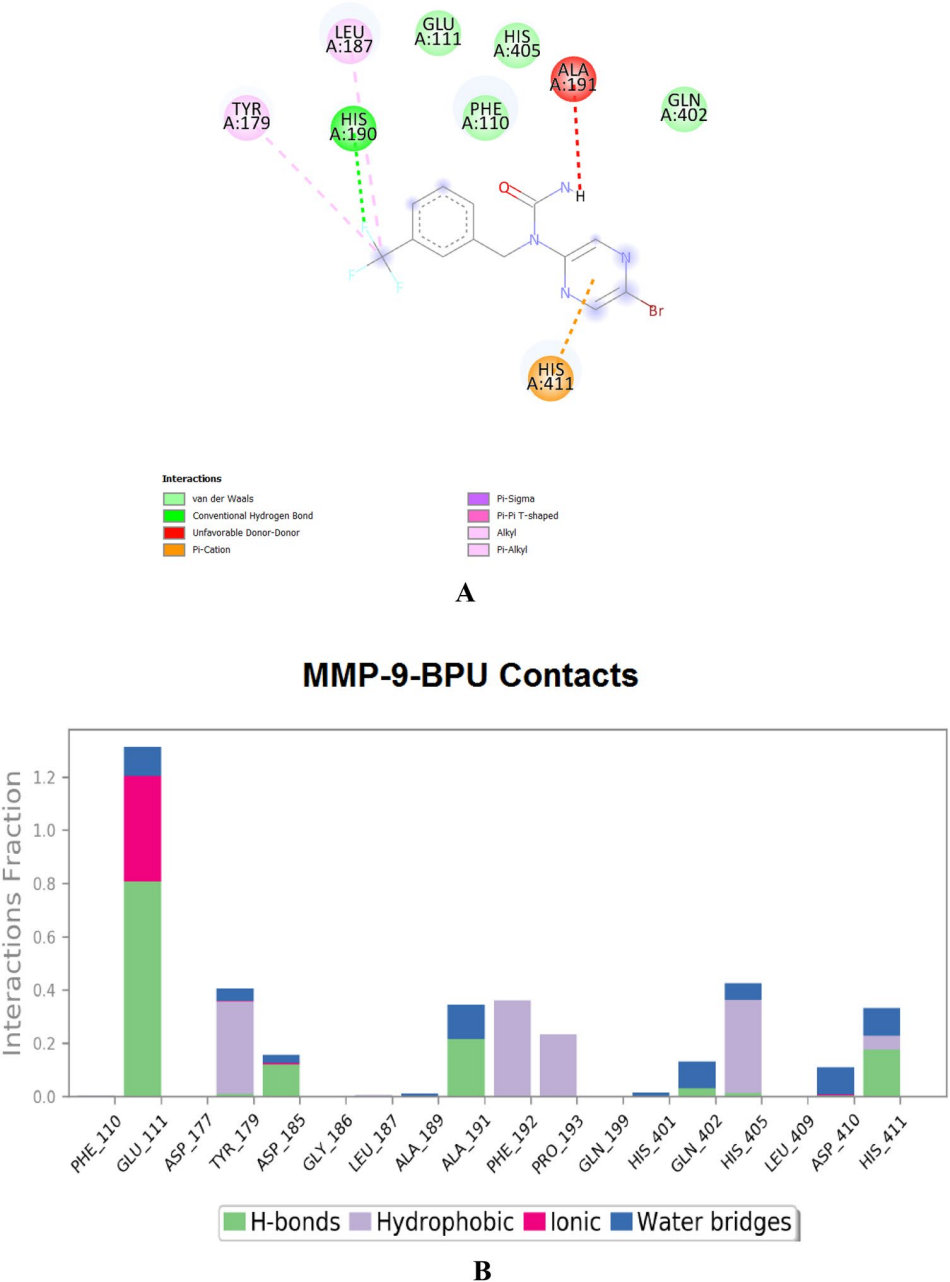
(**A**) Docking molecular interactions of the compound **BPU** with the protein MMP-9 (PDB ID: 2OVX); net binding energy = − 7.8 kcal/mol. (**B**) MMP-9-**BPU** interactions fractions estimated by 100-ns MD simulation.

It is worth mentioning that both enzymes are partially controlled by some elemental ions, e.g., mainly, zinc and calcium ions^20^. Inhibiting both enzymes could be achieved via many binding routes or modes, like binding zinc ions (i.e., demetallation; to disrupt optimal coordination of the catalytic zinc), forming physiologically-unfavorable strong bonds (specially with the active-site cleft to block it), and/or impairing accommodation of the large hydrophobic groups in the slightly flexible catalytic cavities (to prevent these cavities from exhibiting the crucial distinct rotational conformations required for each protein to perform its dynamic/pharmacological roles)^20^. The current computational docking and dynamics findings showed relatively strong inhibitory binding of the **BPU** molecule to the proposed principal catalytic residues of both enzymes (e.g., with Leu82, Leu83, Ala86, Glu130, Pro141, and Tyr143 residues in the case of MMP-2 and with Glu111, His190, Ala191, Gln402, His405, and His411 residues in the case of MMP-9) as well as with the catalytic zinc ions (very good zinc-chelating capacity). The **BPU** molecule also interacts with the catalytic region from Pro421 to Arg424 in the MMP-9 protein, but with very slight interactions that appear only in the three-dimensional (3D) visualizations. The **BPU** molecule showed its largest contact (interaction) with the MMP-2 protein at the Ala86 residue (about 2%), while it showed its largest contact with the MMP-9 protein at the Glu111 residue (above 1.3%). The strong abilities of the ligand **BPU** to form strong hydrogen bonds and intensely sequester zinc ions are mainly attributed to the electron-rich four nitrogen and one oxygen atoms of the pyrazine and urea moieties. The intermolecular interactions (together with their percentages) of **BPU** with each of MMP-2 and MMP-9 are shown in Figs. 9A,B and 10A,B, respectively (zinc ions were removed from docking images to enhance the visibility of the amino acid residues). The computational results of the molecular docking indicated that the **BPU** molecule is able to strongly bind to the MMP-2 enzyme and its identified catalytic domain (i.e., active site) with a good net binding energy of − 9.0 kcal/mol and similarly bind to the MMP-9 enzyme with a good net binding energy of − 7.8 kcal/mol. The reference native ligand of the MMP-2 protein is the aryloxyphenyl-heptapeptide hybrid TP0556351 (acts mainly via its L-peptide linker *N*-methyl-isoleucine “IML”), while the reference native ligand of the MMP-9 protein is the barbiturate inhibitor RO-206–0222 “4MR” (5-(4-phenoxyphenyl)-5-(4-(pyrimidin-2-yl)piperazin-1-yl)pyrimidine-2,4,6(1*H*,3*H*,5*H*)-trione). The **BPU** molecule exhibited better or, at least, comparable interactions with the MMP-2 and MMP-9 enzymes when compared with the two native ligands of the two enzymes (the two native ligands also showed less negative net binding energies, − 8.7 and − 7.1 kcal/mol, with their enzymes, MMP-2 and MMP-9, respectively; see Fig. S3A,B and Fig. S4A,B, respectively, in the complementary Supplementary Material file).

**Figure 11:**
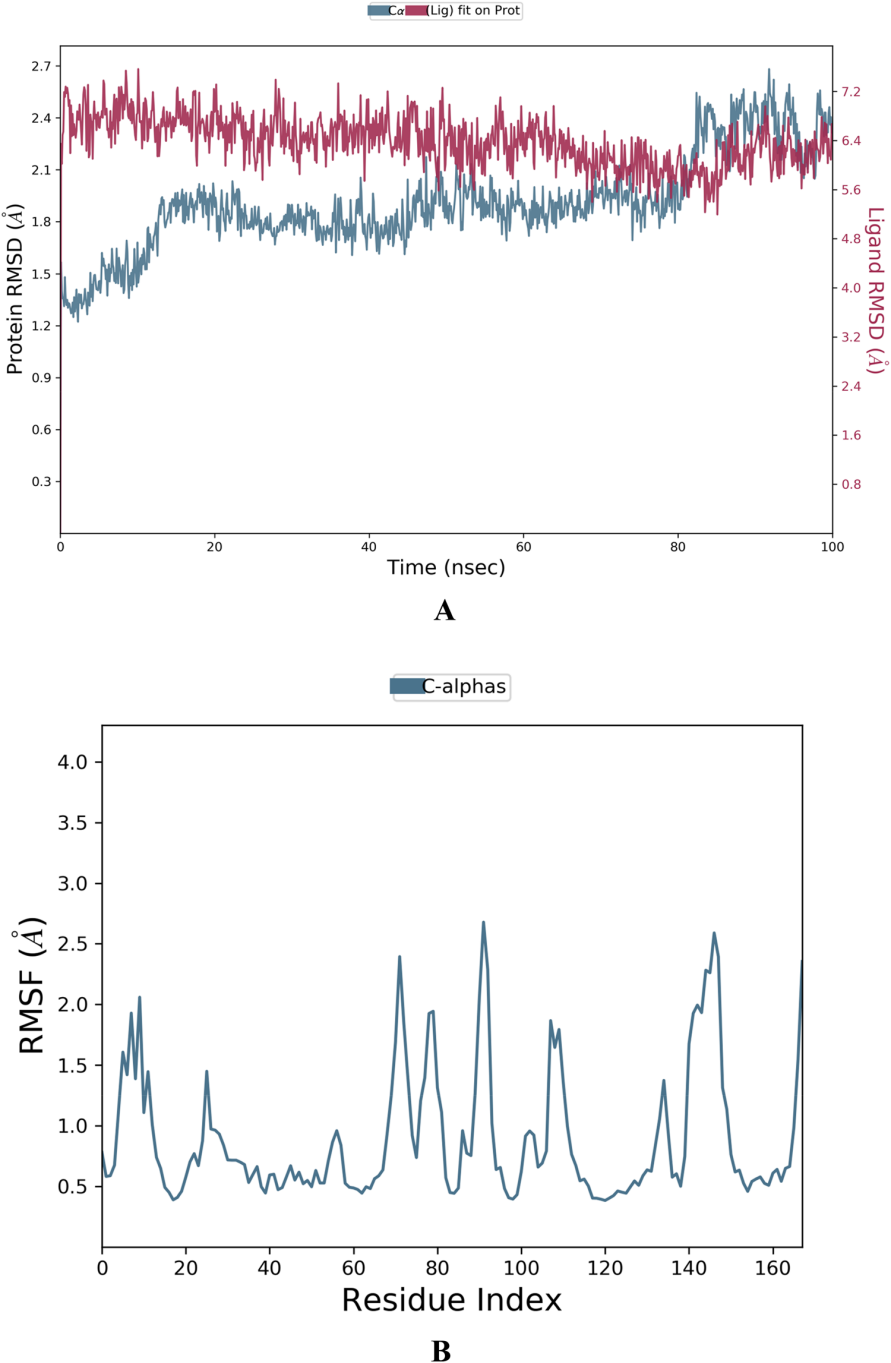
(**A**) RMSD chart outputted during the simulated interaction of the compound **BPU** with the protein MMP-2 (PDB ID: 7XGJ) over 100 ns. (**B**) RMSF chart of the protein MMP-2 (PDB ID: 7XGJ), outputted during the simulated interaction with the compound **BPU**.

**Figure 12:**
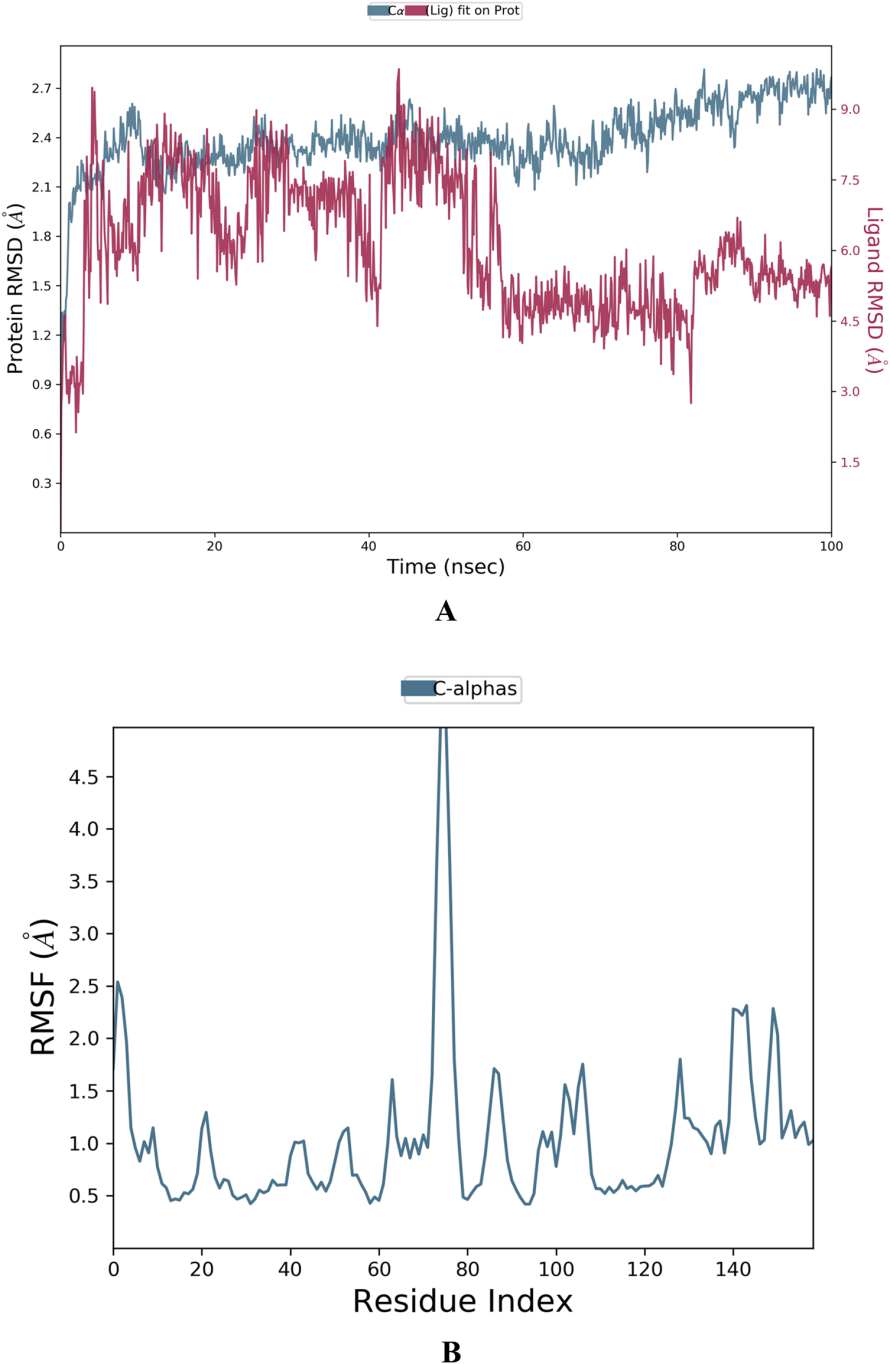
(**A**) RMSD chart outputted during the simulated interaction of the compound **BPU** with the protein MMP-9 (PDB ID: 2OVX) over 100 ns. (**B**) RMSF chart of the protein MMP-9 (PDB ID: 2OVX), outputted during the simulated interaction with the compound **BPU**.

**Figure 13:**
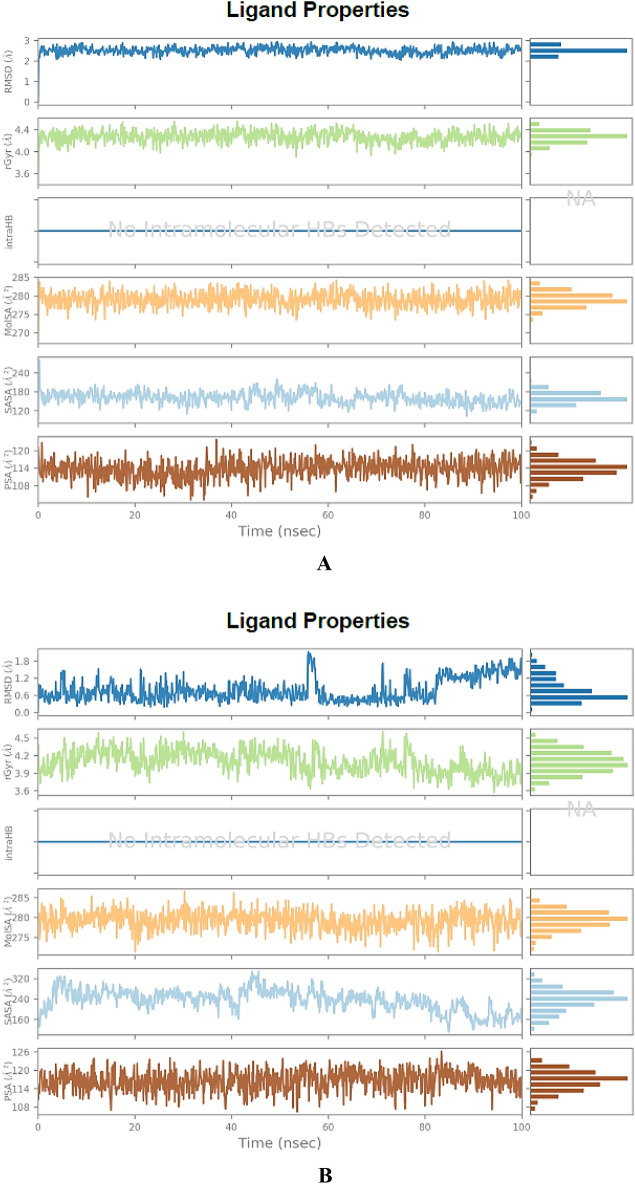
Conformational properties (RMSD, rGyr, intraHB, MolSA, SASA, and PSA) of the potential ligand **BPU**, outputted during the 100-ns simulated interaction of the compound **BPU** with the proteins: (**A**) MMP-2 (PDB ID: 7XGJ). (**B**) MMP-9 (PDB ID: 2OVX).

Bioinformatics tools play a vital role in the analysis of big chemical data. MD simulations frequently make use of Newton's classical equation of motion to calculate atom movements over time. The root-mean-square deviation (RMSD) is used to measure the average alteration in displacement of a group of atoms for a specific frame with regard to a reference frame (typically the first frame is used as the reference and it is regarded as the zero of time). RMSD is calculated for all frames in the trajectory. On the other hand, the root-mean-square fluctuation (RMSF) is very useful for recognizing local modifications along the protein chain (i.e., the protein residues). Interestingly, the **BPU** molecule showed relatively low RMSD/RMSF values with the two proteins MMP-2 and MMP-9 (as clearly noticed in Figs. 11A,B and 12A,B, respectively) when compared with the two native ligands of both proteins (as also clearly noticed in Fig. S5A,B and Fig. S6A,B, respectively, in the complementary Supplementary Material file) over most simulation intervals and residue indices, respectively. The RMSD chart of the outputted MMP-2-**BPU** complex displayed very stable protein–ligand contacts over the entire 100-ns simulation period, as seen in Fig. 11A. On the other hand, the RMSD chart of the outputted MMP-9-**BPU** complex displayed two distinct equal-interval stability patterns of less stable protein–ligand contacts (the first is from 0 ns to about 50 ns, while the second is from about 50 ns to 100 ns, as seen in Fig. 12A) due to structural conformational modification and adaptation over the entire 100-ns simulation period. Other conformational properties, such as the radius of gyration (rGyr), intramolecular hydrogen bonds (intraHB), molecular surface area (MolSA), solvent-accessible surface area (SASA), and polar surface area (PSA), of the candidate ligand **BPU** within the binding active cavities of the two proteins are of relatively good low values and also of more stable values and patterns (as seen in Fig. 13A,B, respectively) when compared with the two native ligands of both proteins (as also seen in Fig. S7A,B, respectively, in the complementary Supplementary Material file) over most simulation intervals, respectively.

The molecular docking and MD simulation findings denoted that **BPU** can efficiently bind to the MMP-2 and MMP-9 proteins with relatively favorable and stable binding interactions (and may potentially successfully inhibit both or one of them, giving very good antitumorigenic activities), and thus can be further explored and employed as a promising potent and selective anticancer agent.

Cancer treatment is one of the important focused areas of interest in current research. Chemoprevention has evolved as a more refined strategy for controlling cancer with fewer side and irritating effects than traditional chemotherapy. However, chemotherapeutic and chemopreventive drugs typically lack multiple targeting ability on cancer cells, are almost nonselective in their actions, and are thus toxic to normal healthy cells and with undesirable side effects. A perfect antitumor drug should target cancer cells in multiple ways (via two synergistic pathways at least) while causing minimal toxicity to normal cells. Since the last few decades, many natural products, like curcumin and others, have been tested and tried for their antitumor potential due to mainly their polyphenolic nature^6^. In parallel, synthetic pyrazine compounds, as previously mentioned in the “Introduction” section, have shown similar inhibitory activities against several malignant cells through multiple pathways while also ensuring cytoprotection against normal cells. For instance, they inhibit the different cell cycle phases in diverse cell lines. Despite the fact that previous antitumor pyrazine derivatives meet the primary criteria for becoming ideal chemopreventive drugs, numerous efforts have been made to overcome limitations for the medicinal use of pyrazine derivatives and also to improve their therapeutic efficacies.

One approach to improve the anticancer capacities and efficiencies of pyrazine compounds is the development of more convenient chemical modifications to their structure with respect to the structure-anticancer activity relationship. **BPU** was designed and successfully synthesized in this context. This compound was evaluated for anticancer activity as well as bioavailability (primarily from a computational viewpoint at the current stage). The ability of anticancer drugs to suppress tumor cell proliferation can be used to determine their efficacy. The MTT assay is a very dependable method for determining cell proliferation and death. Accordingly, the cytotoxicity of the compound **BPU** was studied using the MTT assay on MCF-7, HeLa, and Jurkat cell lines. All the three cancer cell lines showed growth inhibitory effects when exposed to the compound **BPU** at increased different concentrations. In Jurkat cells, among the three cell lines, the compound **BPU** was potent and very efficient, showing about 9 times the potency of its parent compound **BPA**. The compound was also effective against the other cell lines, but to lesser degrees. Additionally, it was discovered that **BPU** is nontoxic with hardly any normal fibroblast cells (NIH-3T3) growth inhibitory effects. Jurkat cells were chosen for further research because, as aforementioned, they responded to the compound **BPU** treatment more sensitively than the other two cell lines evaluated. In order to track the course of the cell cycle, we used flow cytometry. Jurkat cells treated with DMSO as a control displayed a normal cell cycle with a high percentage of cell populations in the G1 phase; however, after 48 h of treatment with the compound **BPU** at its highest dosage (20 µM), cells considerably accumulated in the sub-G1 phase (32.57%). Apoptosis can be detected early by the considerable accumulation of cells in the sub-G1 phase. Our findings indicate that the compound **BPU** induces cancer cell death, resulting in an increased cell population in the sub-G1 phase, which may lead to apoptosis and thus favorably affect cell proliferation. The antiangiogenic effects of **BPU** are also an important factor contributing to its promising comprehensive tumor-regressive action. Upon treatment with **BPU** in both CAM assays (in-vivo and ex-vivo assays), inhibition rates were very significant, measuring about 83% and 77%, respectively, with a substantial decrease of approximately 92% in the total length of the blood vessels in the chicken eggs. In addition, the in-silico docking score of **BPU** was − 9.0 kcal/mol for the MMP-2 enzyme and − 7.8 kcal/mol for the MMP-9 enzyme, with relatively strong hitting and stable simulated binding to the catalytic sites of the two enzymatic proteins, supporting the strong activities of **BPU** against angiogenesis, tumor growth, and metastasis. Consequently, when compared to currently available cancer treatment medicines (present in the literature), the molecule **BPU** exhibits very encouraging binding outcomes with proteins involved in tumors progression.

## Conclusions and future therapeutic plans

**Figure 14:**
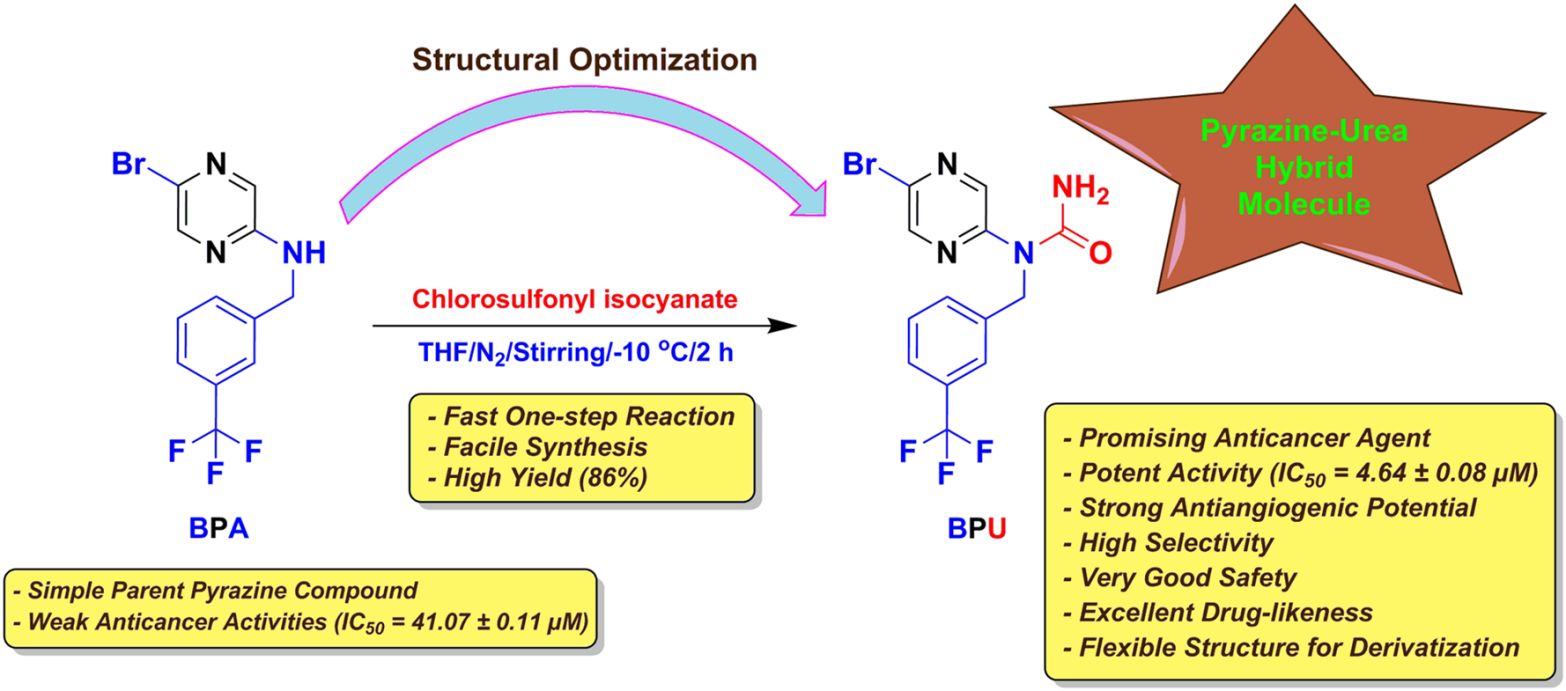
A concluding graphical abstract of the current work.

In our continued trials to create a more effective treatment option for cancer patients, a new candidate drug called **BPU** was synthesized by conducting a substitution reaction on the secondary amino group of the parent weak/moderate anticancer pyrazine compound **BPA**. This was done to potentiate the moderate activity of **BPA** against cancer cells through incorporating the antiproliferative urea moiety in its skeleton. Our present findings suggest that the new pyrazine compound **BPU** could be a promising anticancer agent since it effectively inhibits tumor cell proliferation as well as tumor tissue angiogenesis and can be selected for further diverse and extensive in-vitro and in-vivo investigations. Moreover, the computational drug-like behaviors of **BPU** as well as the computational docking/dynamics scores and molecular interactions of **BPU** with some cancer-progression-involved MMPs are very encouraging. Findings obtained from analyzing the simulated computational interactions suggest that the compound **BPU** might exhibit its anticancer properties and activities, at least partially, via inhibiting the two crucial enzymes MMP-2/MMP-9 using several binding modes, for instance, those affecting the coordination spheres of MMP-2/MMP-9. Consequently, the compound **BPU** shows promising binding results as compared to the relevant existing drugs used for similar cancer inhibition. These results can be further validated, extended, and promoted in well-designed future therapeutic plans by conducting wet-lab analysis so as to develop better and novel synthesized **BPU** analog drugs (i.e., extension for a chemical series of hybrid pyrazine-urea scaffold) for cancer treatment, which will have much less side/adverse effects and higher activities. The most important findings of the current work are summed up and illustrated in Fig. 14.

## Materials and methods

### Chemistry

#### Chemicals and reagents

All chemicals, reagents, and solvents (as well as drugs used for the biological screening) used in the current studies (chemical and biological experiments) were of pure analytical grade and purchased from S.D. Fine-Chem Limited (SDFCL), Mumbai Branch, India, unless otherwise specified.

#### Synthetic procedure for the precursor compound “**BPA**”

To a solution of 5-bromopyrazin-2-amine (purchased from Sigma-Aldrich “Merck”; 1 g, 0.0057 mol) and 3-(trifluoromethyl)benzaldehyde (purchased from Sigma-Aldrich “Merck”; 1 mL, 0.0074 mol) in excess ethyl acetate (up to 100 mL), TFA (0.64 mL, 0.0084 mol) was added and the reaction mixture was stirred for 4 h at R.T. To continue the reductive amination reaction, STAB (3 g, 0.0142 mol) was added to the reaction mixture, and again, the mixture was stirred at R.T. for about 12 h. After this reaction interval, TLC (using hexane/ethyl acetate “4:1” as the eluent system) showed complete consumption of the starting materials and the presence of a sole new spot that corresponds to the product** BPA** in its crude status (as outlined in the scheme of Fig. 2). After completion of the reaction as monitored by TLC, the mixture was quenched with water, extracted with ethyl acetate (3 × 100 mL), and then washed with brine solution. The combined organic layers were dried over sodium sulfate, filtered, and finally concentrated to afford the required almost-pure product of **BPA** in very good yield and with spectroscopic/elemental analyses corresponding to the proposed structure as mentioned below (please see the complementary Supplementary Material file).

Yield: 92%. M.P.: 111–114 °C. IR (KBr, *ν*_max_ (cm^−1^)): 1334 (C=N). ^1^H-NMR (400 MHz, DMSO-*d*_6_, *δ* (ppm)): 4.31 (s, 2H, CH_2_), 5.21 (s, 1H, NH), 7.25–7.43 (m, 4H, 4 Ar–H), 8.22–8.44 (m, 2H, 2 Pyrazine-H). ^13^C-NMR (400 MHz, DMSO-*d*_6_, *δ* (ppm)): 42.62, 123.43, 123.96, 124.81, 128.64, 130.16, 130.94, 131.23, 133.15, 137.81, 143.69, 157.07. LC–MS (*m*/*z*, MW = 332.12): 332.02 ([M]^+^), 334.15 ([M + 2]^+^). Elem. Anal. (%, for C_12_H_9_BrF_3_N_3_): *Calculated*: C, 43.40; H, 2.73; N, 12.65 & *Found*: C, 43.42; H, 2.75; N, 12.68.

#### Synthetic procedure for the designed compound “**BPU**”

Chlorosulfonyl isocyanate was added dropwise to a solution of **BPA** in THF (equimolar amounts “0.01 mol” of both reactants) at − 10 °C under nitrogen atmosphere. The reaction mixture was stirred at the same temperature for 2 h. After these 2 h, the reaction mixture was checked by UPLC. A small amount of the reaction mixture in a vial was quenched with water, and the water layer was then directly loaded in UPLC at R_t_ = 1.66 min, 80% (M^+^ = 375.00, 376.00; UV analysis), which corresponds to product mass. As product formation was confirmed by UPLC, the whole reaction mixture was slowly quenched with water. For working up the product, the pH of the reaction mixture was made neutral, and the mixture was extracted with ethyl acetate (3 × 100 mL) and washed with brine solution thereafter. The combined organic layers were dried over sodium sulfate, filtered, and then concentrated to afford the crude reaction mass of **BPU**. The crude mass of **BPU** was purified by Grace column chromatography, and the product was eluted at 60% ethyl acetate in petroleum ether to afford the required pure product of **BPU** in very good yield and with spectroscopic/elemental analyses corresponding to the proposed structure as mentioned below (please see the complementary Supplementary Material file).

Yield: 86%. M.P.: 123–125 °C. HPLC Purity: > 96%. IR (KBr, *ν*_max_ (cm^−1^)): 1335 (C=N). ^1^H-NMR (400 MHz, DMSO-*d*_6_, *δ* (ppm)): 4.95 (s, 2H, CH_2_), 6.46 (s, 2H, NH_2_), 7.26–7.52 (m, 4H, 4 Ar–H), 8.40–8.44 (s, 2H, 2 Pyrazine-H). ^13^C-NMR (400 MHz, DMSO-*d*_6_, *δ* (ppm)): 52.68, 123.32, 124.54, 125.72, 128.68, 129.45, 130.30, 130.94, 133.91, 137.21, 140.25, 156.44, 158.46. LC–MS (*m*/*z*, MW = 375.15): 375.01 ([M]^+^), 377.05 ([M + 2]^+^). Elem. Anal. (%, for C_13_H_10_BrF_3_N_4_O): *Calculated*: C, 41.62; H, 2.69; N, 14.93 & *Found*: C, 41.71; H, 2.74; N, 14.98.

### Biological studies

Three different types of cancer cell lines, MCF-7 (human breast cancer cell line), HeLa (human cervical cancer cell line), and Jurkat (human T lymphocyte cell line) cells, were selected for the preliminary cytotoxic screening of the compounds **BPA** and **BPU** to assure diversity of the cancer cells examined with respect to type and location. In addition, these three types are almost the most famous cell lines used for research purposes during the evaluation journeys of potential anticancer agents. Specifically, the 3-(4,5-dimethylthiazol-2-yl)-2,5-diphenyltetrazolium bromide (MTT) assay^21,22^ was employed to assess the growth inhibitory potential of the compounds **BPA** and **BPU** with few modifications. Further, analysis of progression of cell cycle was performed by flow cytometry^5,23^ to confirm the mechanism of induction of cell death in the **BPU**-treated Jurkat cells. To explore the antiangiogenic potential of the final target compound, **BPU**, and confirm its capacities against tumor invasion, the in-vivo/ex-vivo CAM assays were also performed. All values of the biological findings were considered statistically significant at *p* < 0.05 and *p* < 0.01.

#### Cell lines and culture

MCF-7, HeLa, and Jurkat cells were purchased from the National Center for Cell Science, Pune, India. Cells were grown in RPM1 1640 supplemented with 10% heat-inactivated fetal bovine serum (FBS), 100 U/mL of penicillin, and 100 µg/mL of streptomycin, and then incubated at 37 °C in a humidified atmosphere with 5% CO_2_. The standard anticancer drugs used for the different types of cell lines are Tamoxifen (MCF-7 cells), Avastin (HeLa cells), and Abitrexate (Jurkat cells).

#### MTT assay

Prior to designing, synthesizing, and extensively evaluating the cytotoxic/antitumor activities of the target compound of the current study, the compound **BPU**, the parent compound **BPA** was preliminarily subjected to an in-vitro evaluation to determine its effectiveness in inhibiting the growth of cancer cells. Three types of cancer cells, namely MCF-7, HeLa, and Jurkat cells, were chosen as targets for this assessment, as above-mentioned. The evaluation was performed using the MTT assay, a common method used for measuring cell viability and proliferation. During the experiment, the compound **BPA** was tested at different concentrations, specifically 5, 10, and 20 µM, and at different time intervals of 48 and 72 h. The cytotoxicity studies were conducted by exposing the cancer cells to the compound **BPA** (according to the steps of the similar experiment mentioned below for the new analog **BPU**) and monitoring the resulting cell death or reduction in proliferation. The results of the experiment were recorded and tabulated, showing the various degrees of cytotoxicity exhibited by the compound **BPA** against the different cancer cell lines. The recorded data allowed for a comprehensive analysis of the effectiveness of the compound in inhibiting the growth of cancer cells.

Later, the cytotoxic effects of the newly-targeted compound **BPU** against the cervical carcinoma, breast carcinoma, and leukemic cells (5 × 10^5^ cells) were extensively assessed using the MTT assay. The test compound was dissolved in DMSO, and then the targeted cells were treated with different increasing concentrations of the test compound (5, 10, and 20 µM, respectively). Cells in the control wells received the same volume of medium containing DMSO. After 48 and 72 h of treatment, cells were harvested and incubated with MTT (0.5 µg/mL) for 4 h at 37 °C in 96-well plates. The blue MTT formazan precipitate formed in the viable cells in each case was solubilized by the addition of 70 µL of DMSO. The suspension was placed in a microvibrator for 5 min, and absorbance was measured at 540 nm using a multimode reader (Varioskan Flash Multimode, Thermo Scientific, USA) for each case. A parallel control cell line of NIH-3T3 cells (with **BPU** administered) was employed to assess the safety and nontoxicity of the compound **BPU** on noncancerous normal cells. The entire experiment was performed in triplicates (repeated at least three times).

#### Cell cycle analysis using fluorescence-activated cell sorter (FACS) technique

Jurkat cells were seeded in a 24-well plate at 0.75 × 10^5^ cells/mL of complete growth culture media, and then they were incubated for 24 h. After incubation, the cells were treated with different concentrations (in DMSO) of the test compound **BPU** (5, 10, and 20 µM) as well as with the pure control solvent DMSO. After 48 h, the cells were effectively harvested, washed with 1X PBS, and then fixed in 70% ethanol at 4 °C overnight. Prior to carrying out flow cytometry, cells were washed with 1X PBS and resuspended in 300 µL of 1X PBS. RNase (50 µg/mL) (Sigma Aldrich, USA) treatment was accurately given. Cells were subsequently stained with propidium iodide (Sigma Aldrich, USA) and finally subjected to flow cytometry (Beckman Coulter, USA) using the CellQuest Pro software, with excitation at 488 nm laser and emission at 560/670 nm. At the end, the DNA content of 10,000 cells was accurately recorded per sample, and histograms were consequently analyzed by the known Flowing software (version 2.5, licensed version).

#### Assessment of the antiangiogenic potential of **BPU** in the shell-less chick chorioallantoic membrane (CAM) angiogenesis assays

The in-vivo/ex-vivo shell-less chick CAM assays are a well-established technique to study tumor angiogenesis/invasion and evaluate the antiangiogenic potential of various compounds^1^. To perform this technique, fertilized chicken eggs (10-day-old chicken eggs; this age period sufficiently provides a range of developmental stages for the CAM and blood vessel formation, which is crucial for conducting CAM assays) were obtained and incubated in a humidified incubator at 37 °C for 2 days, allowing the embryos to develop. On day 2, the eggs were carefully cracked open over a sterile cup, releasing the contents into the cup without damaging the embryos. The embryonic material, consisting of the CAM and yolk sac, was gently placed in sterile petri dishes, taking care not to rupture blood vessels. The petri dishes were then covered and returned to the incubator under ideal incubation conditions. On day 4 of development, a CAM was treated with the compound being tested, in this case **BPU**, at a 1 mg/mL concentration. A CAM treated with only PBS was used as a normal-developing control. The rVEGF_165_ alone was used as a placebo negative control (0% angiogenesis inhibition) in an untreated CAM, while a CAM treated with the relevant standard concentration (1 mg/mL) of Cisplatin was used as a reference positive control. To stimulate angiogenesis, filter discs soaked in the growth factor rVEGF_165_ were carefully placed on the CAMs. The dishes were reincubated, and after 72 h, the extent of vascularization and antiangiogenic response was evaluated. The newly-formed blood vessels in treated and control CAM assays were visualized, photographed using a digital camera (a high-quality steady-shot Sony DSC-W610 camera), and quantified to determine the effect of the test compound **BPU** on angiogenesis. Multiple images were acquired to ensure a comprehensive analysis. This shell-less CAM test allowed direct visualization and quantification of the antiangiogenic potential of **BPU**.

### In-silico studies

#### Protein preparation

The 3D crystalline structures of the targeted proteins (two enzymes) were retrieved from the Protein Data Bank “PDB” (http://www.rcsb.org). The retrieved protein PDB IDs 7XGJ (the MMP-2 enzyme) and 2OVX (the MMP-9 enzyme) were chosen to be used for docking with the target ligand **BPU**. These crystallized forms of the two proteins were specifically chosen for the current investigation due to their previous high validation, the similarity of their cocrystallized inhibitors (at the active domains) with the currently-investigated compound** BPU**, and also due to their use in previous similar studies. The coordinates of the structures were complexed with water molecules and other elemental atoms/ions as needed^23–25^. The 3D resolution and quality were increased as much as possible (along with carrying out any requested addition/removal of certain water molecules/elemental atoms) using the Discovery Studio software^23–25^. Finally, the respective files were saved in the .*pdb* format for further processing.

#### Ligand preparation and energy minimization

The two-dimensional (2D) and 3D chemical structures of the ligand **BPU** (chosen after obtaining its promising anticancer activities from the biological experiments, while the parent ligand **BPA** was excluded from the computational investigation due to its relatively low anticancer biological activities) were accurately drawn using the known ChemDraw software^25–27^. The simplified molecular-input line-entry system (SMILES) notation format was used primarily to draw both structures of **BPU**, which then converted into the *.mol* file using the same ChemDraw software. Ligand structure was energy minimized using the universal force field (UFF) in the ArgusLab software and then saved in the *.pdb* format for further processing^23–27^. Further, the Gasteiger charges were added to the ligand structure, which was made flexible using the respective tools of AutoDock Vina software and finally saved in the *.pdbqt* format^23,24,26^.

#### Binding pocket analysis

Binding pocket/cavity plays a key role in the bioactive binding interaction between the targeted receptor and chemical ligand in any drug design process. The binding pockets/cavities in the currently-targeted two proteins were considered default with respect to their native complexed inhibitors^23–27^. The pharmacophoric selection was carried out using the AutoDock Vina tools, and each cavity consisted of all the listed pharmacophores from the crystallized data^23–27^. Finally, the protein with the selected cavity size/dimensions was saved in the *.pdbqt* format in each case prior to the blind docking^23–27^.

#### Ligand–protein docking

The current in-silico molecular docking was carried out using the AutoDock Vina (version 1.2.3, licensed version) tools, 10 conformations (poses) of ligands for each complex were generated, and the complex with the lowest energy (highest score) was considered. Exactly as with the best docking pose selection, validation of the docking process is also one of the most important points in the in-silico docking studies. In the current case herein, we validated the docking process by interacting with the indigenous/native inhibitor of each targeted protein and comparing the pharmacological interaction (the in-silico binding) with the crystallized data represented on the PDB website with respect to the least-energy complex data generated (i.e., validation through the redocking procedures)^28,29^. Later, we used the same detected cavity of each protein as the principal catalytic active site, docked **BPU** in the protein, and finally listed the in-silico pharmacological interaction for the least-energy complex generated in each case^28,29^.

#### Molecular dynamics (MD) simulations

One hundred nanoseconds of MD simulation were described by Desmond (Schrodinger, Inc.) for each produced docking complex. The simulations of the receptor and ligand complexes (for both enzymes with the target compound **BPU** and with the specific reference “native” ligand of each enzyme in each time) were initiated by the docking experiments, as previously mentioned. In static situations, molecular docking studies may forecast a ligand's binding state, giving a static view of the molecule's binding posture at the protein's active site. MD simulations were utilized to predict the ligand's binding status in a physiological environment. The receptor-ligand complexes were preprocessed using Maestro's Protein Preparation Wizard, which included complex optimization and reduction. The system was set up using the System Builder tool. It was decided to use the orthorhombic box TIP3P (Transferable Intermolecular Interaction Potential 3 Points) solvent model. The OPLS 2005 force field was utilized for the simulations. Counter ions were added to the mixture to neutralize the models. The addition of 0.15 M sodium chloride (NaCl) almost simulated physiological conditions. Throughout each simulation, the NPT ensemble was utilized, with a temperature of 310 K and a pressure of 1 atm. The models were relaxed before the simulation. All the other steps were implemented exactly as instructed/recommended in the software's manual. The stability of the simulation was assessed by contrasting the RMSD of the protein and ligand over time. Trajectories were saved for examination at 100-ps intervals of each 100-ns simulation period. Using Maestro's Simulation Interaction Diagram, various post-simulation analyses were carried out and reported in the current work.

#### ADMET screening

Here we calculated the ADMET properties for the target molecule **BPU** using the two famous online webservers SwissADME (http://www.swissadme.ch) and admetSAR 2.0 (http://lmmd.ecust.edu.cn/admetsar2) to predict the significant pharmaceutical and toxicological properties. Physicochemical properties, such as molecular formula, molecular weight (MW), heavy atoms number, aromatic heavy atoms number, rotatable bonds number, hydrogen bond (H-bond) acceptors/donors numbers, total polar surface area (TPSA), lipophilicity values, water solubility, some other several pharmacokinetics parameters, and Lipinski's rule violations number, were calculated from the SwissADME server^28,30^. On the other hand, other pharmacokinetic and toxicological items, such as Caco-2 cell permeability, blood/brain barrier, human intestinal absorption, carcinogenicity, and acute oral toxicity, were calculated using the admetSAR server^31^.

### Supplementary Information

Supplementary Information 1.

Supplementary Information 2.

Supplementary Information 3.

Supplementary Information 4.

Supplementary Information 5.

## Data Availability

All data generated or analyzed during this study are included in this published article and its supplementary information (Supplementary Material and Supplementary MD Simulation) files.
