# Supplementary material for: Discovery of 1-(5-bromopyrazin-2-yl)-1-[3-(trifluoromethyl)benzyl]urea as a promising anticancer drug via synthesis, characterization, biological screening, and computational studies
Source: Sci Rep. 2023 Dec 20;13:22824. doi: 10.1038/s41598-023-44662-x (PMC10739849; doi:10.1038/s41598-023-44662-x)
Supplement: Supplementary file 1 — Supplementary Information 1. [file 41598_2023_44662_MOESM1_ESM.pdf]

Supplementary Material

Discovery of 1-(5-Bromopyrazin-2-yl)-1-[3-(trifluoromethyl)benzyl]urea as a Promising Anticancer Drug *via* Synthesis, Characterization, Biological Screening, and Computational Studies

Authors' Names & Affiliations:

Yasser Hussein Issa Mohammed[1,2,], Israa M. Shamkh[3,4], Nahed S. Alharthi[5], Mohammed A. Shanawaz[6], Hind A. Alzahrani[7], Basit Jabbar[8], Saba Beigh[9], Saad Alghamdi[10], Nada Alsakhen[11], Elshiekh B. Khidir[12], Hayaa M. Alhuthali[13], Taqwa Hafiz Elamin Karamalla[14], and Amgad M. Rabie[15,*]**

[1] Department of Biochemistry, Faculty of Applied Science, University of Hajjah, Hajjah, Yemen
[2] Department of Pharmacy, Faculty of Medicine and Medical Science, University of Al-Razi, Sana'a, Yemen (Email: issayasser16@gmail.com, ORCID iD: 0000-0003-1086-7292)
[3] Botany and Microbiology Department, Faculty of Science, Cairo University, Giza, Egypt
[4] Chemo and Bioinformatics Lab, Bio Search Research Institution (BSRI), Giza, Egypt (Email: israamshamkh@gmail.com)
[5] Department of Medical Laboratory Sciences, College of Applied Medical Sciences in Al-Kharj, Prince Sattam Bin Abdulaziz University, Al-Kharj 11942, Saudi Arabia (Email: naahamursy@hotmail.com)
[6] Department of Public Health, Faculty of Applied Medical Sciences, Albaha University, Albaha 65431, Saudi Arabia (Email: mshenwaz@bu.edu.sa)
[7] Department of Basic Sciences, Faculty of Applied Medical Sciences, Albaha University, Albaha 65431, Saudi Arabia (Email: dhndzahrani@bu.edu.sa)
[8] Centre of Excellence in Molecular Biology, University of the Punjab, Lahore 53700, Pakistan (Email: basit.ibb.pu@gmail.com)
[9] Department of Public Health, Faculty of Applied Medical Sciences, Albaha University, Albaha 65431, Saudi Arabia (Email: beigh.sabba@gmail.com)
[10] Department of Clinical Laboratory Sciences, Faculty of Applied Medical Sciences, Umm Al-Qura University, Makkah, Saudi Arabia (Email: ssalghamdi@uqu.edu.sa, ORCID iD: 0000-0003-4532-9128)
[11] Department of Chemistry, Faculty of Science, The Hashemite University, Zarqa, Jordan (Email: nada-alsakhen@hu.edu.jo)
[12] Department of Clinical Laboratory Sciences, Faculty of Applied Medical Sciences, Umm Al-Qura University, Makkah, Saudi Arabia (Email: ebkhidr@uqu.edu.sa)
[13] Department of Clinical Laboratory Sciences, College of Applied Medical Sciences, Taif University, P.O. Box 11099, Taif 21944, Saudi Arabia (Email: mhuthal@tu.edu.sa)
[14] Faculty of Medicine, Helwan University, Helwan, Cairo, Egypt (Email: taqwahafizelamin@gmail.com)
[15] Head of Drug Discovery & Clinical Research Department, Dikernis General Hospital (DGH), Magliss El-Madina Street, Dikernis City 35744, Dikernis, Dakahlia Governorate, Egypt (E-mail: amgadpharmacist1@yahoo.com, ORCID iD: 0000-0003-3681-114X)

***** ***Principal Corresponding Author***:

Dr. Amgad M. Rabie

E-mail: amgadpharmacist1@yahoo.com, ORCID iD: 0000-0003-3681-114X

****** ***Second Corresponding Author***:

Dr. Yasser Hussein Issa Mohammed (E-mail: issayasser16@gmail.com, ORCID iD: 0000-0003-1086-7292)

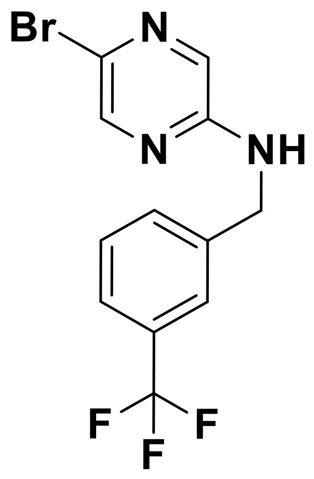

Fig. S1. Chemical structure of the newly-synthesized chemical intermediate **BPA**.

Chart S1: ^{1}H-NMR Chart of BPA:

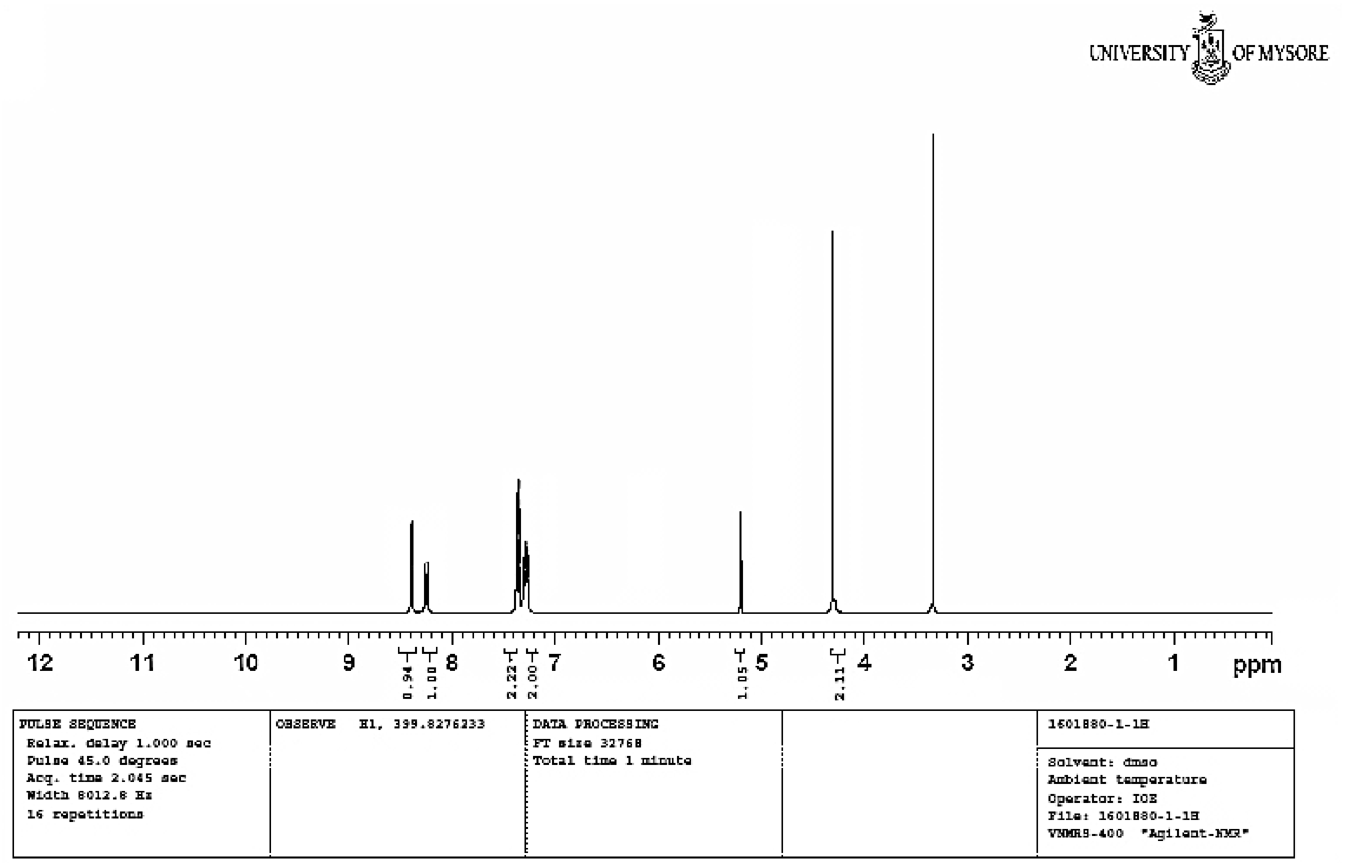

Chart S2: ^{13}C-NMR Chart of BPA:

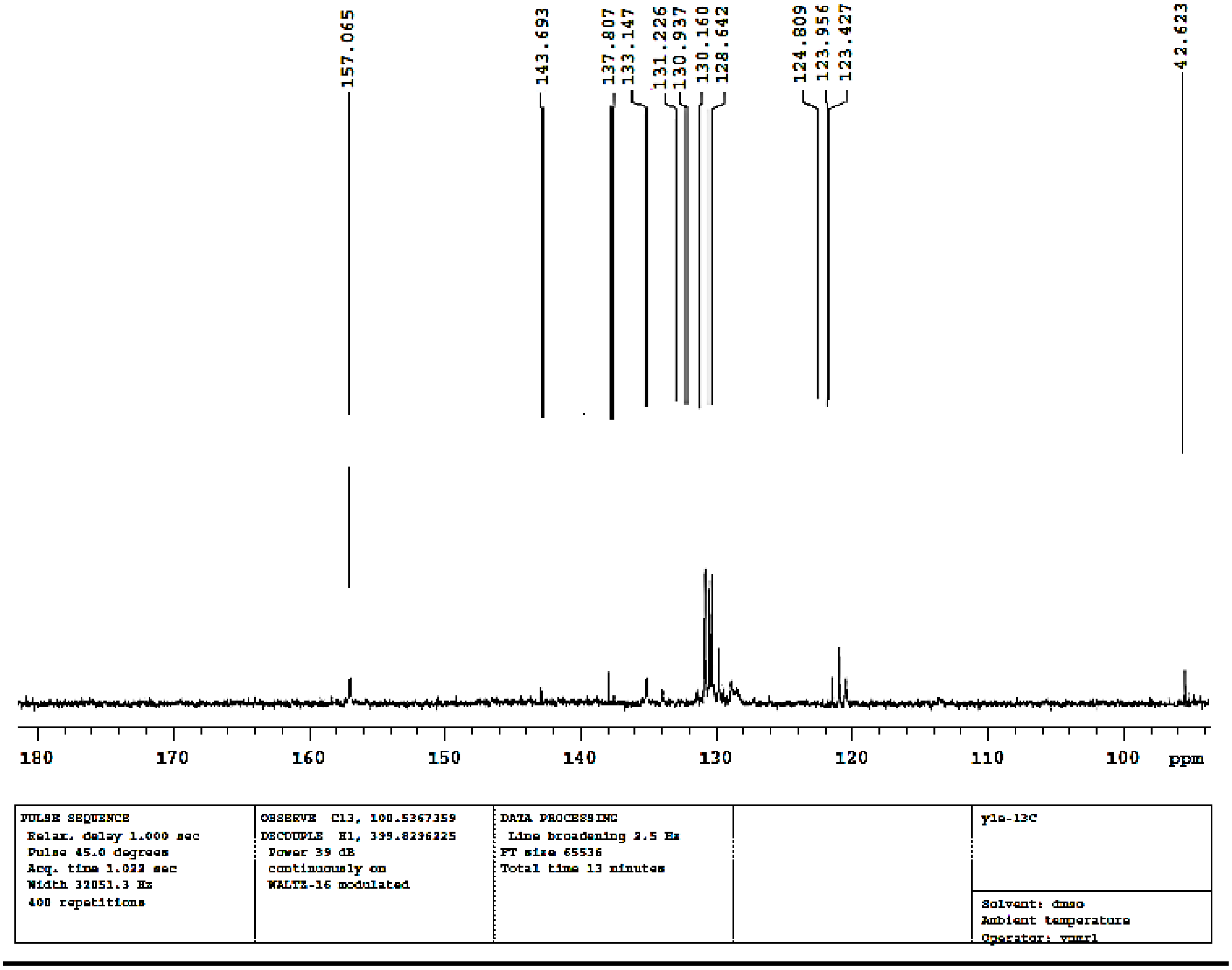

Chart S3: LC-MS Chart of BPA:

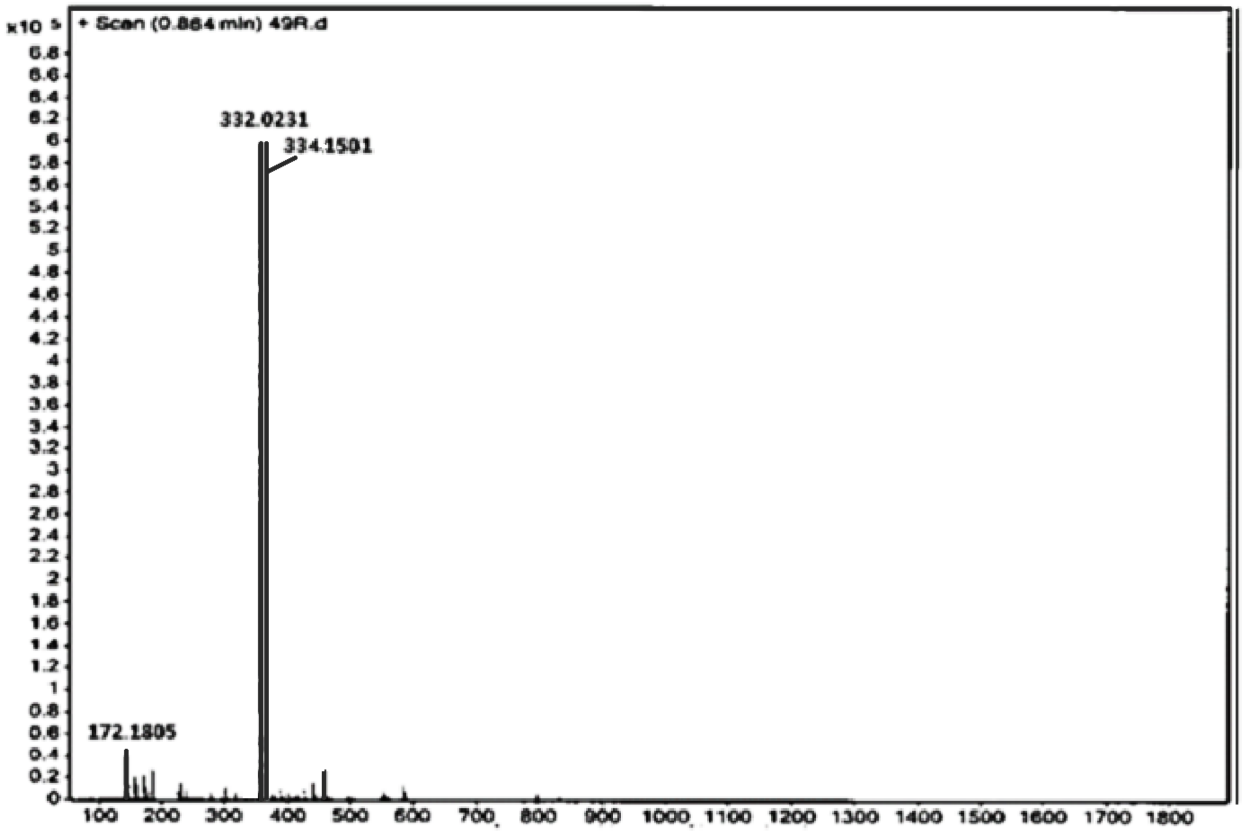

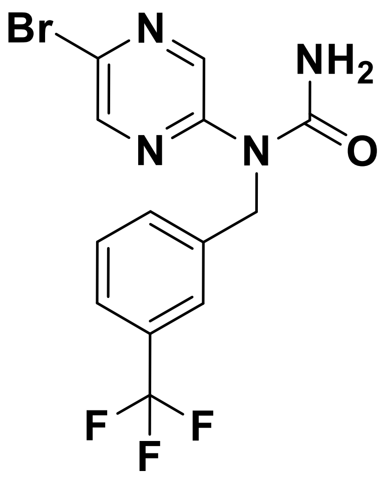

Fig. S2. Chemical structure of the newly-synthesized promising anticancer agent **BPU**.

Chart S4: HPLC Chart of BPU:

```
Data File C:\CHEM32\1\DATA\CYTXON 2021-08-16 12-55-20\021-0301.D
Sample Name: H-6

=====================================================================
Acq. Operator   : Anusha                        Seq. Line :   3
Acq. Instrument : hplc-001                       Location : Vial 21
Injection Date  : 16-Aug-21 1:32:19 PM                Inj :   1
                                               Inj Volume : 5.0 µl
Different Inj Volume from Sequence !     Actual Inj Volume : 20.0 µl
Acq. Method     : C:\CHEM32\1\DATA\CYTXON 2021-08-16 12-55-20\CYTXON.M
Last changed    : 16-Aug-21 1:44:26 PM by Anusha
                  (modified after loading)
Analysis Method : C:\CHEM32\1\METHODS\PRAJNA\CYTXON.M
Last changed    : 16-Aug-21 1:30:52 PM by Anusha
                  (modified after loading)
Additional Info : Peak(s) manually integrated
```

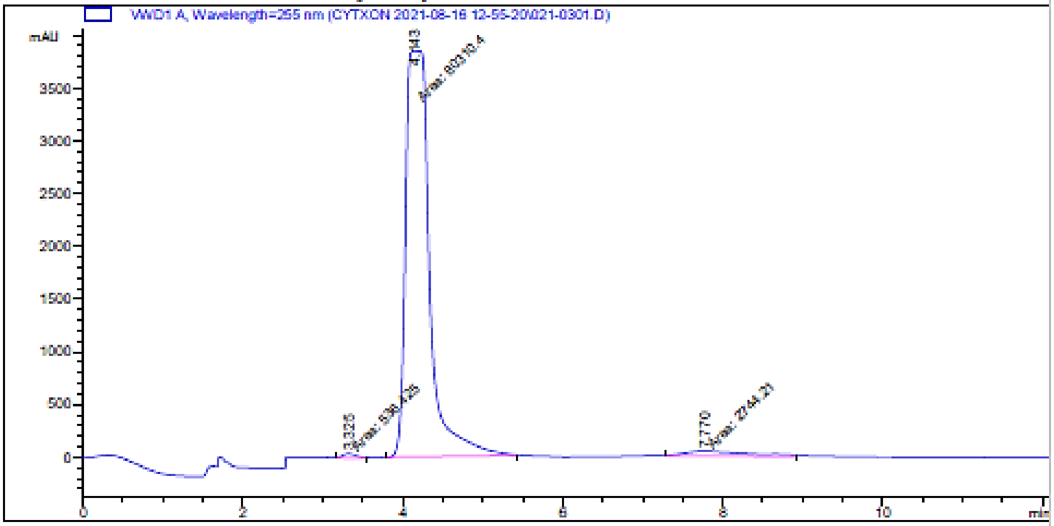


```
=====================================================================
                      Area Percent Report
=====================================================================

Sorted By             :      Signal
Multiplier:                  :      1.0000
Dilution:                    :      1.0000
Use Multiplier & Dilution Factor with ISTDs


Signal 1: VWD1 A, Wavelength=255 nm
```

Peak #	RetTime [min]	Type	Width [min]	Area mAU *s	Height [mAU]	Area %
1	3.325	MM	0.2097	536.42456	42.62711	0.6417
2	4.143	MM	0.3482	8.03104e4	3844.57715	96.0754
3	7.770	MM	0.9947	2744.21289	45.98076	3.2829
Totals :				8.35910e4	3933.18502	

Chart S5: ^{1}H-NMR Chart of BPU:

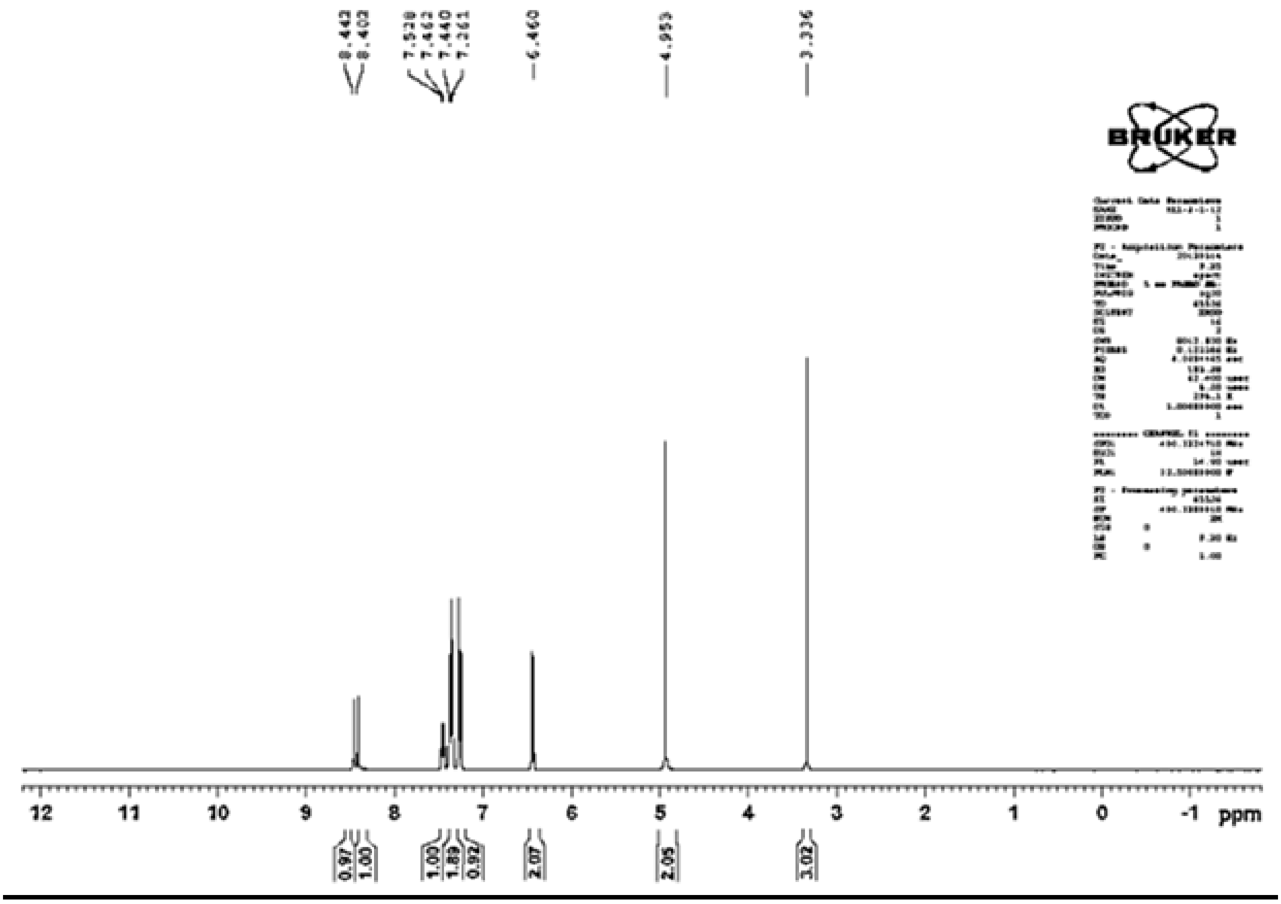

<u>Chart S6: ^{13}C-NMR Chart of BPU</u>:

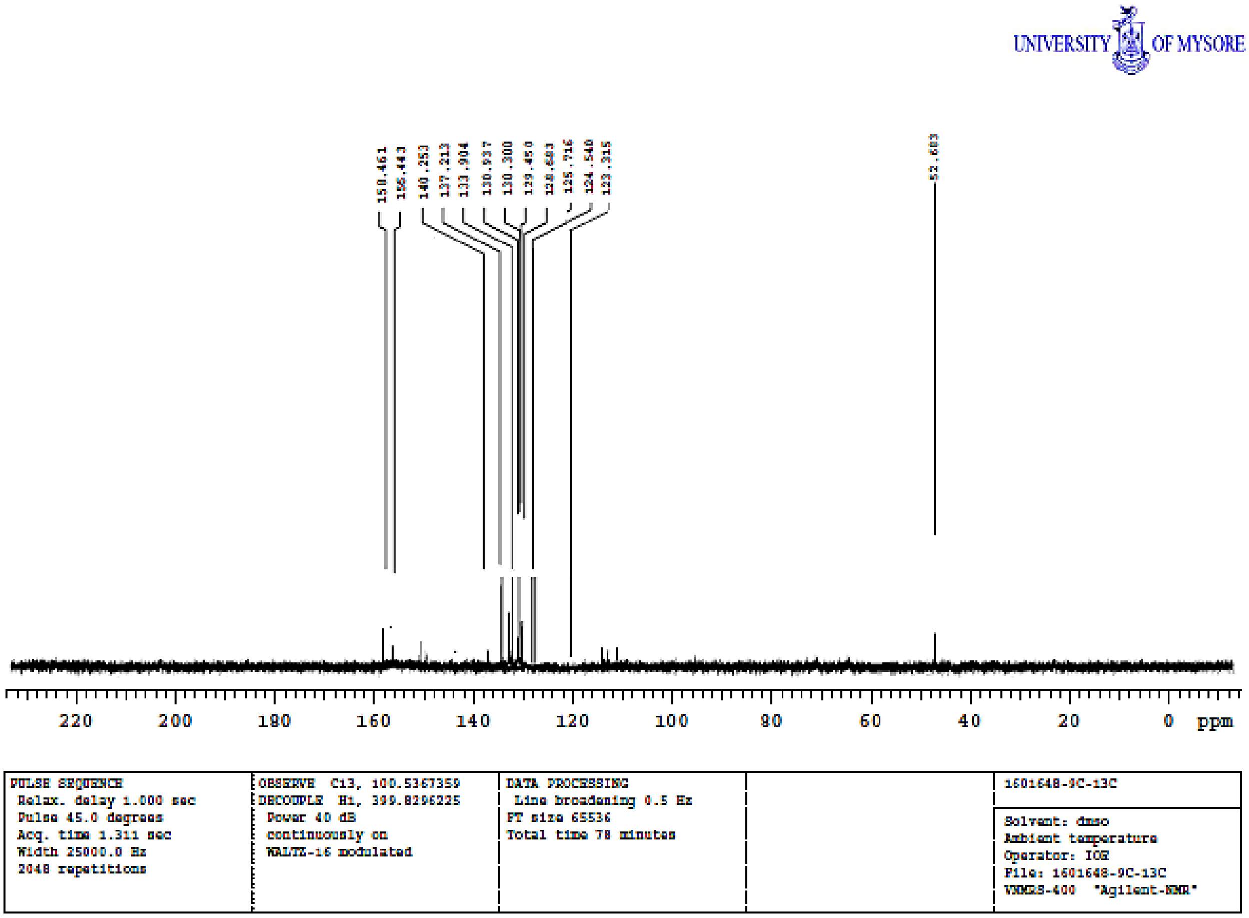

Chart S7: LC-MS Chart of BPU:

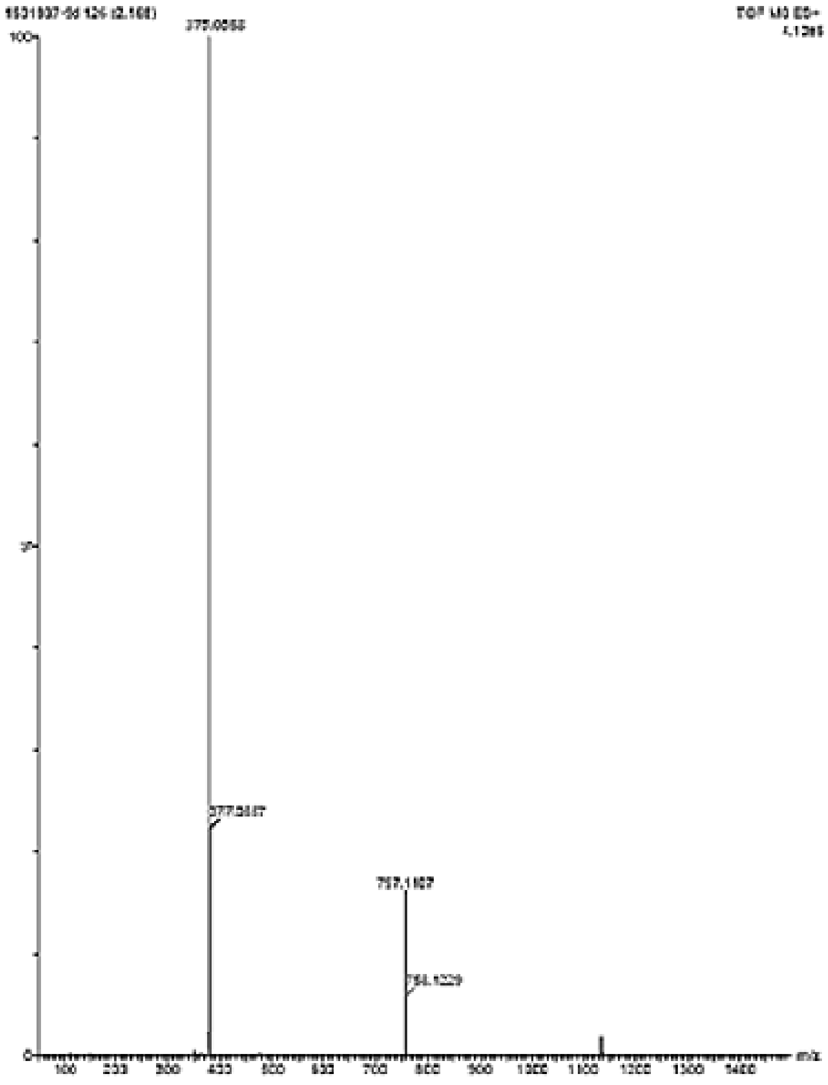

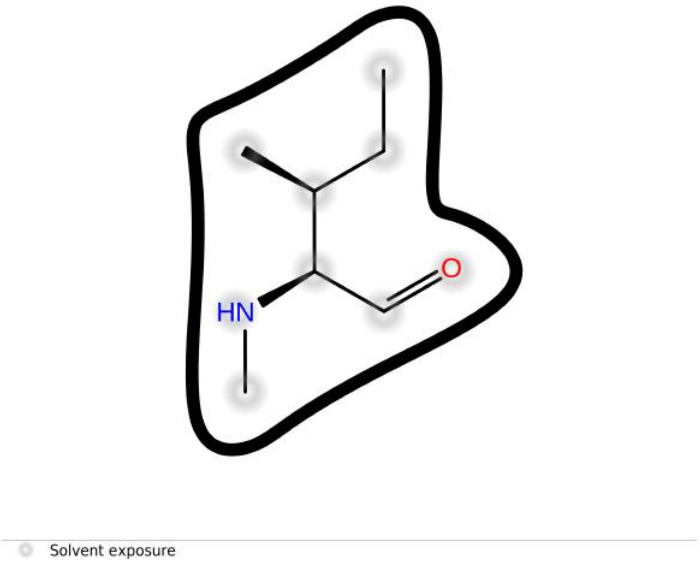

A

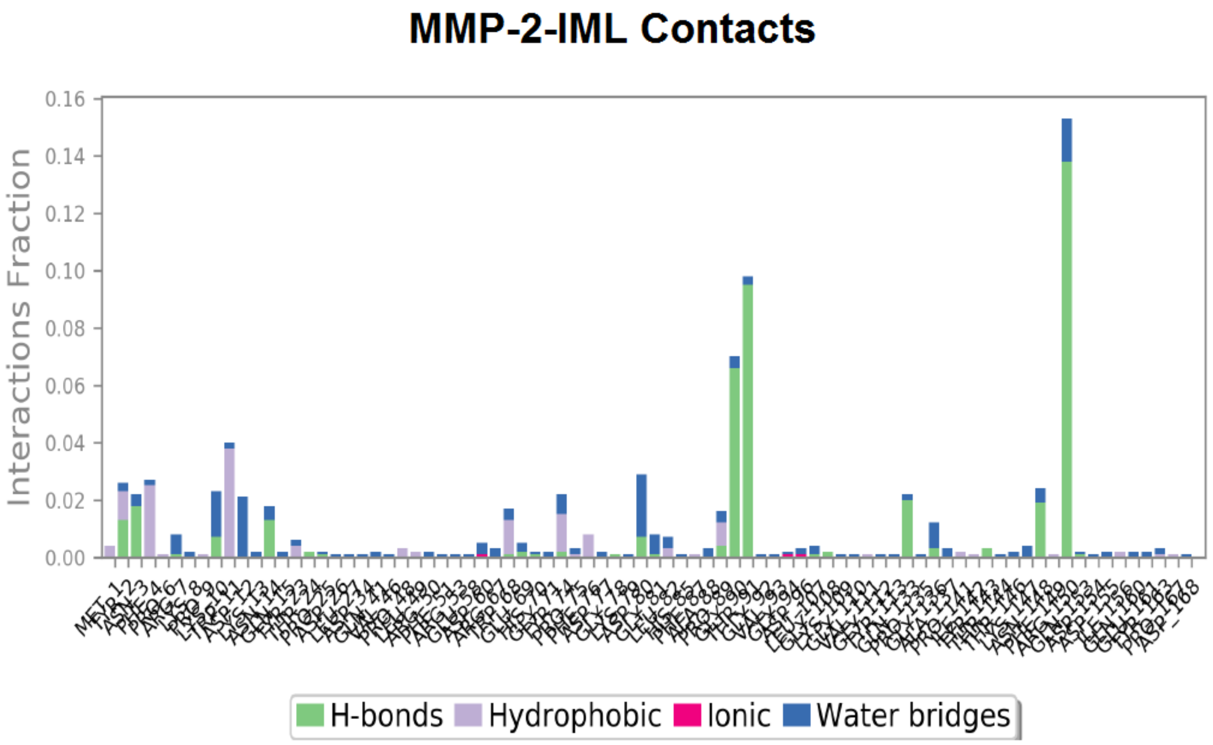

B

Fig. S3. (A) Docking molecular interactions of the native ligand IML with its protein MMP-2 (PDB ID: 7XGJ); net binding energy = -8.7 kcal/mol. **(B)** MMP-2-IML interactions fractions estimated by 100-ns MD simulation.

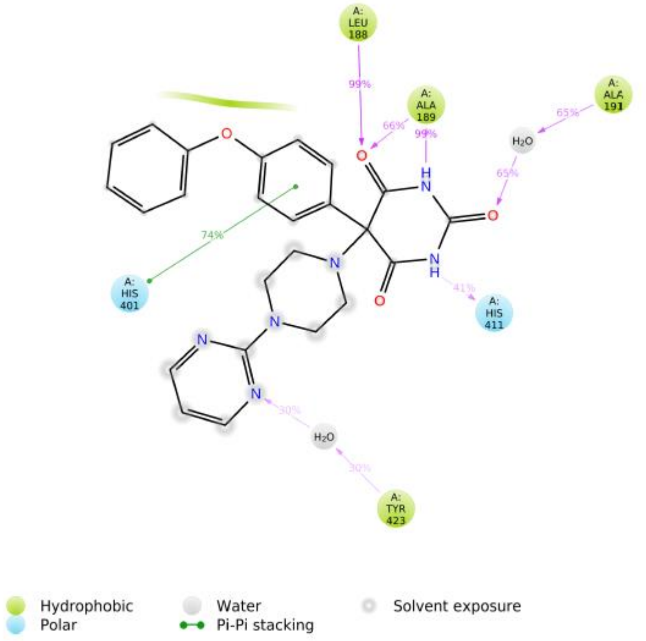

A

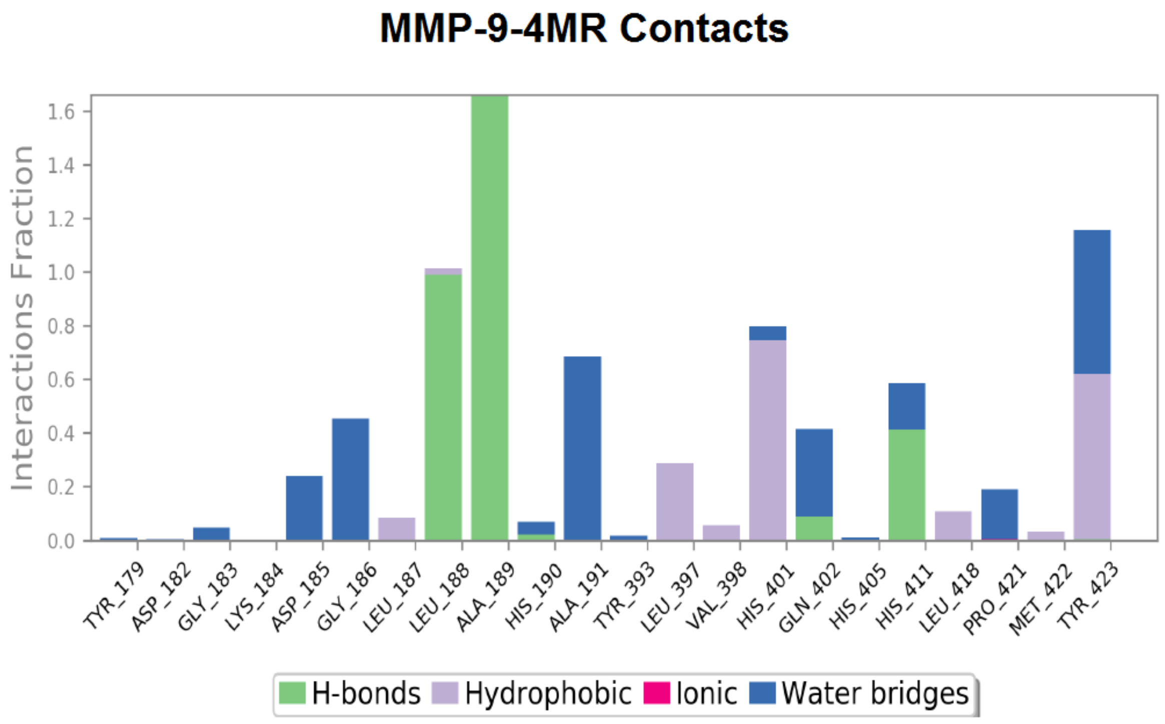

B

Fig. S4. (A) Docking molecular interactions of the native ligand 4MR with its protein MMP-9 (PDB ID: 2OVX); net binding energy = -7.1 kcal/mol. **(B)** MMP-9-4MR interactions fractions estimated by 100-ns MD simulation.

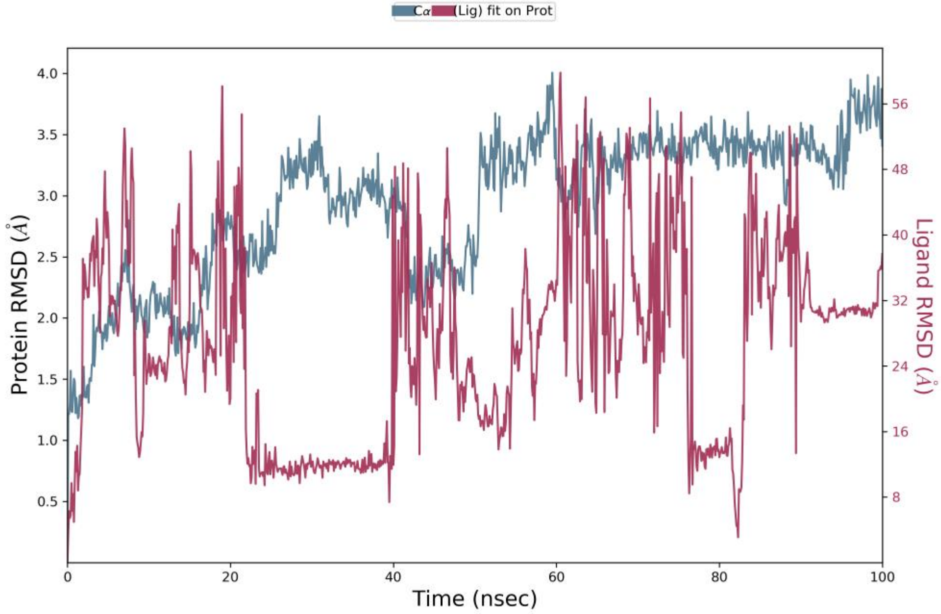

A

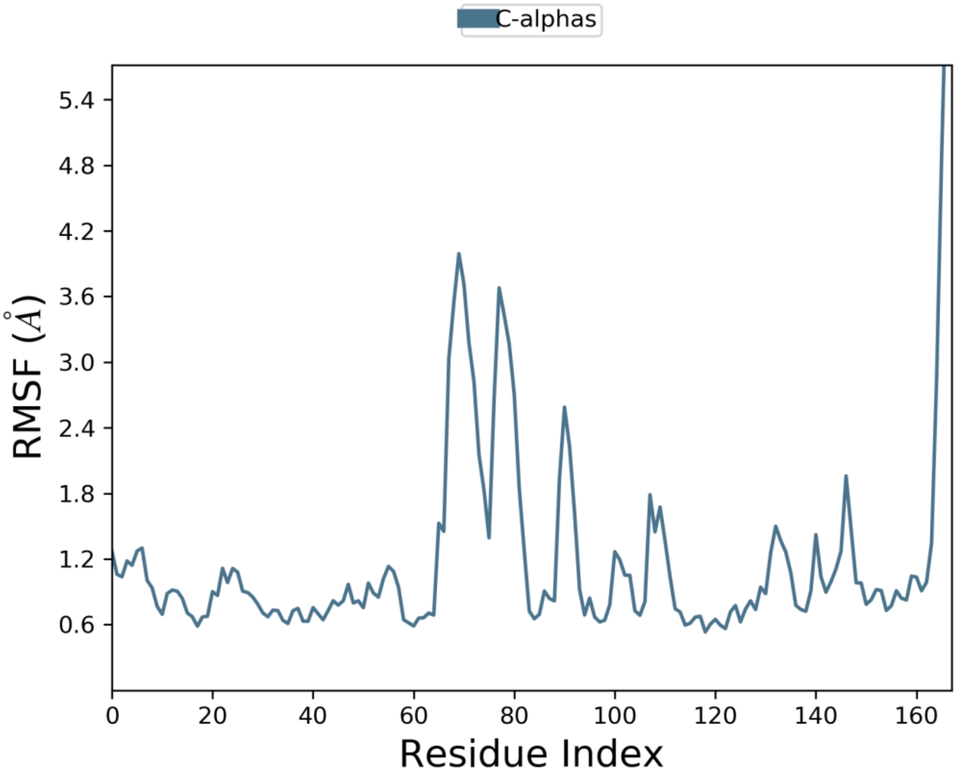

B

Fig. S5. (A) RMSD chart outputted during the simulated interaction of the native ligand IML with its protein MMP-2 (PDB ID: 7XGJ) over 100 ns. **(B)** RMSF chart of the protein MMP-2 (PDB ID: 7XGJ), outputted during the simulated interaction with the native ligand IML.

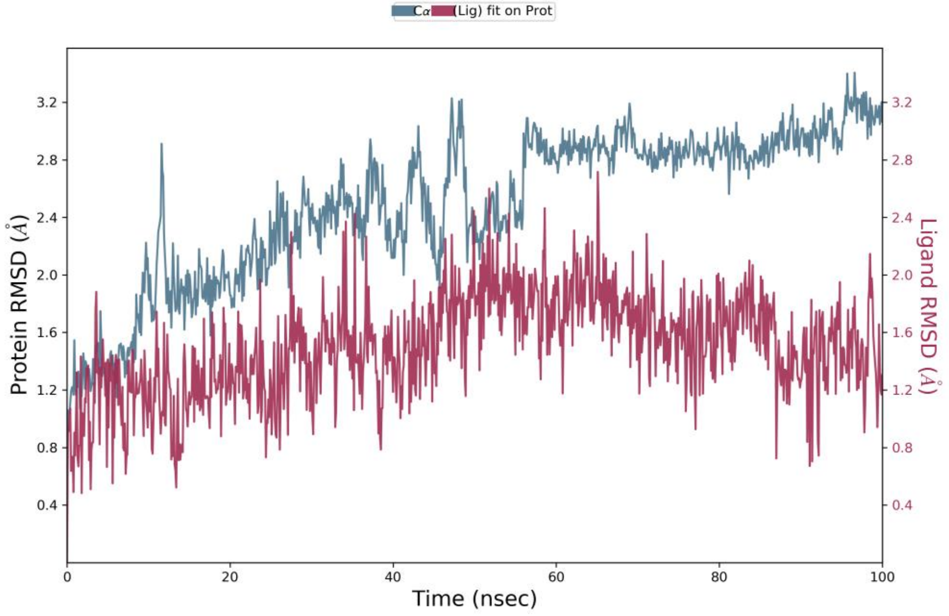

A

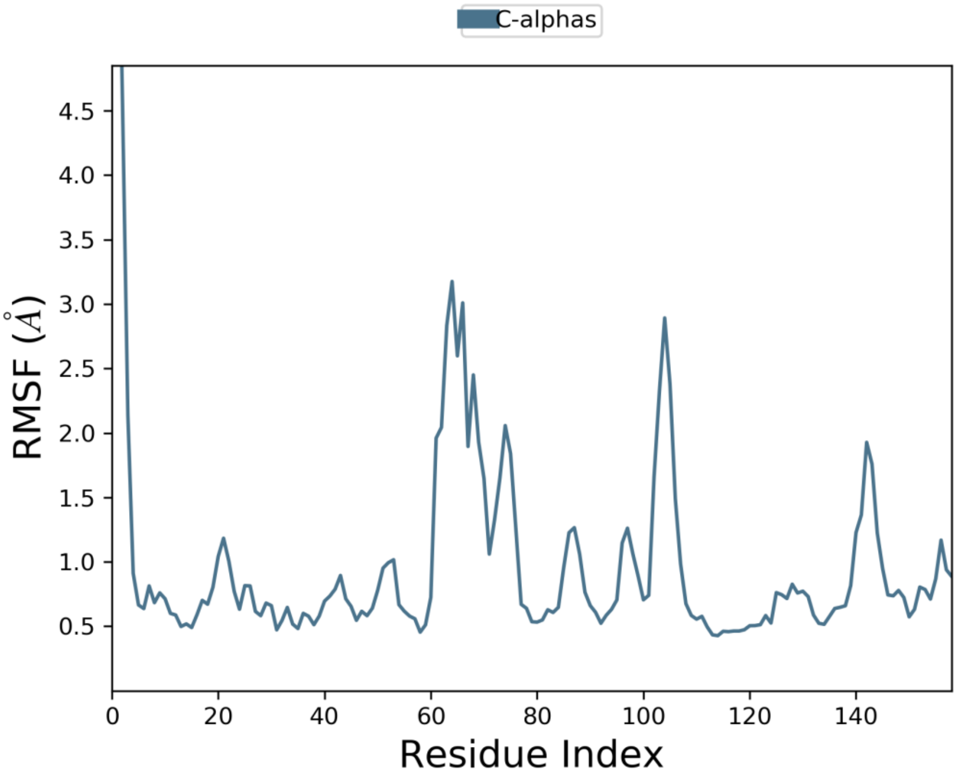

B

Fig. S6. (A) RMSD chart outputted during the simulated interaction of the native ligand 4MR with its protein MMP-9 (PDB ID: 2OVX) over 100 ns. **(B)** RMSF chart of the protein MMP-9 (PDB ID: 2OVX), outputted during the simulated interaction with the native ligand 4MR.

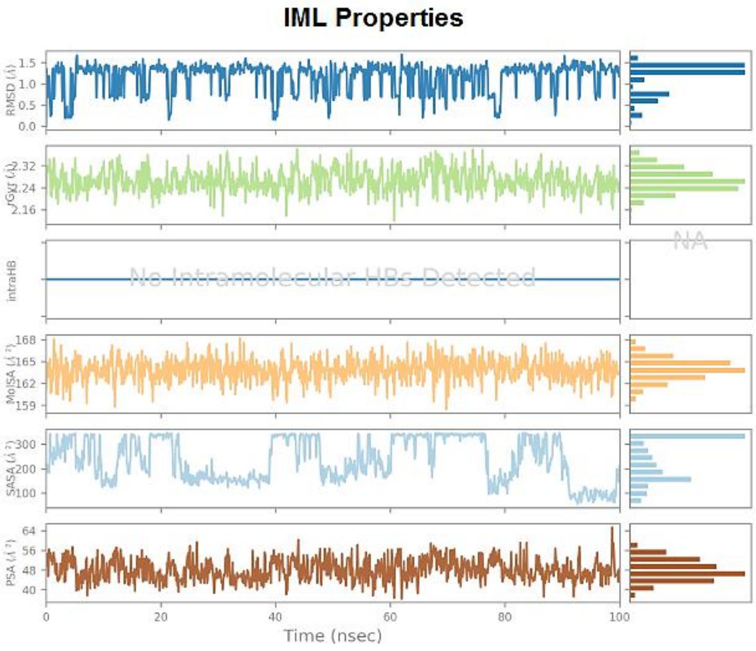

A

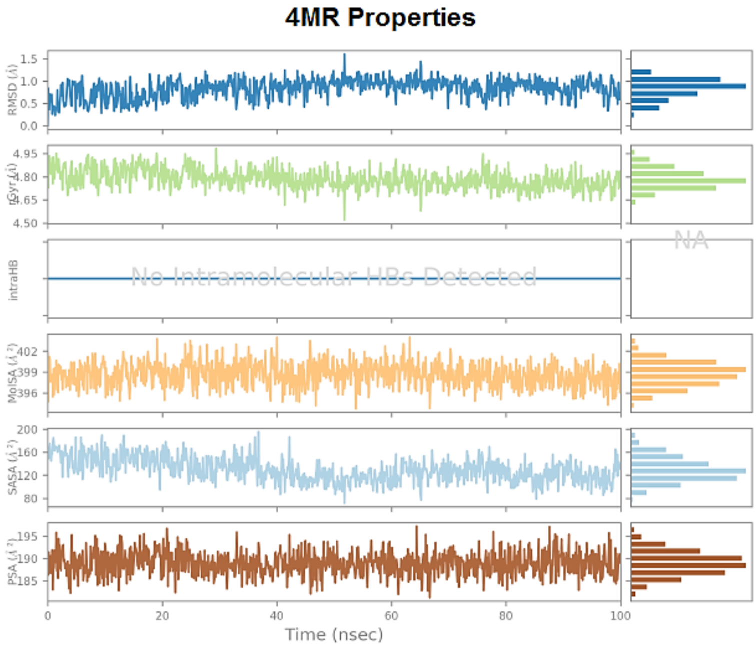

B

Fig. S7. (A) Conformational properties (RMSD, rGyr, intraHB, MolSA, SASA, and PSA) of the native ligand IML, outputted during the 100-ns simulated interaction of this ligand with its protein MMP-2 (PDB ID: 7XGJ). **(B)** Conformational properties (RMSD, rGyr, intraHB, MolSA, SASA, and PSA) of the native ligand 4MR, outputted during the 100-ns simulated interaction of this ligand with its protein MMP-9 (PDB ID: 2OVX).
